# Blockchain-Assisted Secure Distributed UAV Localization Under Byzantine and False Data Injection Attacks

**DOI:** 10.3390/s26216980

**Published:** 2026-11-04

**Authors:** Md Kamrul Islam, Md Mainul Islam, Jannatul Bushra Orpa, Joobeom Yun

**Affiliations:** 1 Department of Computer and Information Security and Department of Convergence Engineering for Intelligent Drone, Sejong University, Seoul 05006, Republic of Korea; kamrul@sju.ac.kr; 2 Department of Electrical and Computer Engineering, Texas A&M University, College Station, TX 77843, USA; mdmainul11@tamu.edu; 3 Department of Computer Engineering, Kwangwoon University, Seoul 01897, Republic of Korea; jannatul@kw.ac.kr

**Keywords:** UAV localization, permissioned blockchain, ADMM, Byzantine attack, false data injection, resilient distributed optimization, smart contract

## Abstract

Cooperative localization lets unmanned aerial vehicles (UAVs) fuse absolute and relative measurements, but the message exchange of distributed solvers is exposed to compromised UAVs. We study distributed weighted-least-squares (WLS) localization with the alternating direction method of multipliers (ADMM) under Byzantine deception, disruption, false data injection (FDI), and adaptive attacks. To mitigate these attacks, we propose a blockchain-assisted ADMM in which a permissioned blockchain records one signed state per UAV and iteration, which prevents equivocation, and a relative-measurement check with a calibrated threshold screens the accepted states; a symmetric edge update makes the method independent of UAV labels. When honest neighbors accept each other, the method converges once the screening decisions stabilize and reject every compromised state, and at any fixed point its deviation from the accepted honest-subnetwork WLS solution is bounded in proportion to the screening threshold. In 1000-UAV simulations with 100–300 compromised UAVs, the proposed defense reduces the FDI error from 23.6–69.9 m to 0.49–0.95 m and stays within 0.03 m of the ideal WLS estimate under equivocating attacks, whereas an adaptive attacker that attempts to evade the threshold causes errors of 3.4–8.5 m. On a six-node Geth Clique network, blockchain-integrated runs reproduce the simulations to within 2 µm, and with a raised block gas limit, an iteration with 1000 submitting UAVs takes about 3.8 s.

## 1. Introduction

Unmanned aerial vehicles (UAVs) are increasingly used in surveillance, disaster response, precision agriculture, package delivery, search and rescue, environmental monitoring, and other cooperative autonomous missions [1,2,3,4], and UAV swarms further enable cooperative sensing, coordinated task execution, and distributed operation in large-scale aerial missions [3,5]. In these applications, accurate localization is essential for navigation, motion planning, formation control, collision avoidance, and task coordination [6,7], and localization errors propagate to higher-level decisions.

Practical multi-UAV localization cannot rely on a single sensing source. Global Navigation Satellite System (GNSS) measurements provide absolute positions but are degraded by noise, multipath propagation, blockage, spoofing, or jamming [8,9]. Cooperative localization therefore combines noisy absolute priors with relative inter-UAV measurements such as range, bearing, ultra-wideband ranging, or displacement [10,11,12,13]. This fusion can be posed as a weighted-least-squares (WLS) problem, whose solution is the maximum-likelihood estimate under Gaussian noise; a centralized solver, however, requires a fusion center, which is a communication bottleneck and a single point of failure. Distributed solvers, in which each UAV computes locally and exchanges information only with its neighbors, are therefore attractive [14,15,16,17]. The alternating direction method of multipliers (ADMM) is well suited to this setting because it decomposes the coupled problem into local subproblems and enforces agreement between neighbors through dual variables [18,19,20,21].

However, the iterative message exchange of a distributed solver also creates an attack surface. A compromised UAV may send different states to different neighbors in the same iteration (equivocation), vary its falsified states over time, or broadcast one consistent but physically incorrect state [22,23,24,25,26]. Such manipulations bias the primal and dual updates, increase the localization error, or prevent convergence. Countering them requires two distinct properties, namely message consistency, which ensures that all honest neighbors use the same value from a given sender, and physical plausibility, which limits the influence of a consistent value that contradicts the measured geometry.

Message consistency is difficult to enforce in a peer-to-peer exchange. Digital signatures authenticate the sender of each message, but a compromised UAV can still sign different states for different neighbors. Receivers can detect such conflicts only by comparing the messages they have received, which requires common neighbors or additional message rounds, as in Byzantine agreement protocols [22]. A permissioned blockchain provides an alternative that does not depend on the communication topology. Its validators agree on a single ordered ledger of records; hence, every UAV reads the same record from a given sender, and a conflicting second record is detected and remains attributable to its sender. Permissioned blockchains achieve this with authenticated participants and without the computational cost of public proof-of-work systems [27,28,29,30]. A blockchain cannot, however, verify that a recorded state is physically correct, because a compromised UAV can always commit one false value consistently. Physical plausibility therefore requires a second check against the relative measurements, which bounds, but cannot eliminate, the influence of false values that remain below its detection threshold.

Motivated by these observations, this paper uses a permissioned blockchain as the primary defense of the ADMM message exchange and complements it with a screening of the accepted states against the relative measurements; the *proposed defense* denotes this combination. The main contributions of this paper are summarized below.

- We specify a threat model for distributed ADMM in cooperative UAV localization that includes receiver-dependent Byzantine deception (equivocation), time-varying disruption, false data injection (FDI), and an adaptive attacker that attempts to evade the screening, and we identify which of these attacks a blockchain can counter.
- To mitigate these threats, we propose a blockchain-assisted ADMM in which a permissioned blockchain accepts one signed state record per UAV and iteration, which prevents equivocation and makes conflicting submissions attributable independently of the communication topology, and in which each UAV screens the accepted states against its relative measurements after a short warm-up period. A symmetric edge update, in which each UAV keeps its own copy of every incident edge subproblem and applies only its own screening decisions, makes the iteration independent of UAV labels and of computations performed by compromised neighbors.
- We show that the method coincides with standard ADMM in the absence of attacks and rejections and that, when honest neighbors accept each other, it converges to the WLS solution of the accepted honest subnetwork once the screening decisions stabilize and reject every compromised state and deviates at any fixed point from that solution by, at most, a bound proportional to the screening threshold. We further show that the screening residual contains the estimation errors of both UAVs and derive a false-alarm bound near convergence that motivates the warm-up period; in the 1000-UAV network with 200 compromised UAVs, the warm-up reduces the false-alarm rate from 54% to 2.1%.
- We implement the protocol on a six-validator Geth Clique network, where the blockchain-integrated runs reproduce the simulations to within 1.7 µm and a load test with up to 1000 submitting UAVs shows that raising the block gas limit and broadcasting the records to all validators reduces the iteration time for 1000 UAVs from 19.3 s to 3.8 s. In 1000-UAV simulations, the proposed defense reduces the FDI error from 23.6–69.9 m to 0.49–0.95 m, mainly through the screening; the blockchain reduces the remaining error under equivocation from 0.47–0.99 m to 0.37–0.45 m; and an adaptive attacker that attempts to evade the threshold still causes errors of up to 3.4–8.5 m, the main remaining vulnerability of the screening.

Section 2 reviews related work, and Section 3 introduces the background. Section 4 formulates the problem and the threat model, Section 5 presents the framework and its properties, Section 6 and Section 7 describe the setup and the results, Section 8 discusses the role and the latency of the blockchain, and Section 9 concludes this paper.

## 2. Related Work

This section positions the proposed framework within four lines of related work.

**Cooperative and distributed UAV localization:** Ruan et al. [10] proposed a coalition formation game for cooperative relative localization in GNSS-denied environments. Yang et al. [11] and Zeng et al. [12] developed ultra-wideband and inertial localization for confined environments and landing platforms, and Du et al. [13] proposed resonant-beam relative localization for swarms. More recent work considers reconfigurable intelligent surface-assisted distributed localization [31], set-membership distributed localization [32], communication-efficient received-signal-strength localization [33], GNSS-free cooperative fusion [34], and UAV–UGV subterranean navigation [35]. These methods improve accuracy, robustness to sensing conditions, or communication efficiency, but they assume that the exchanged optimization messages are honest.

**Security-aware localization:** Michieletto et al. [8] combined GNSS, inertial, and received-signal-strength information for secure navigation of UAV formations under GNSS spoofing. Zhong et al. [9] studied Byzantine distributed detection of GPS spoofing, and Liu et al. [36] studied robust topology recovery for the detection and localization of electronic signals. These works address spoofed sensing or damaged topologies rather than manipulated messages inside an iterative distributed solver.

**Resilient consensus and Byzantine-resilient distributed optimization:** A large body of work protects iterative distributed algorithms against Byzantine agents without any shared ledger. The weighted mean-subsequence-reduced (W-MSR) algorithm discards, at every node, up to *F* extreme neighbor values and guarantees resilient asymptotic consensus on sufficiently robust graphs [37]. Sundaram and Gharesifard [38] and Su and Vaidya [39] characterized what distributed optimization can achieve when some agents are adversarial, and trimmed or robust aggregation schemes extend these ideas to decentralized learning [40,41,42]. These methods do not require trusted infrastructure, but they rely on graph-robustness conditions and filter values by rank rather than by physical consistency, and they cannot prevent equivocation by themselves. Our framework is complementary to these methods, because the blockchain removes equivocation and records conflicting submissions, and a measurement-based check bounds the influence of the remaining consistent false values. We use the W-MSR rule as a baseline in Section 7.

**Blockchain for UAV security:** Blockchains have been used for UAV communication, authentication, clustering, data delivery, and consensus management [43,44,45,46,47,48,49]. These studies secure identities, data sharing, or network management. None of them uses a blockchain to accept the states exchanged inside a localization solver, and none combines such a rule with a physical-consistency check of bounded effect on the estimate.

Table 1 summarizes the most closely related technical studies; its columns distinguish Byzantine resilience in the sense of the cited works from the two mechanisms combined in this paper, namely a non-equivocation layer and a check of physical consistency against measurements. For the proposed method, Byzantine resilience holds within the threat model of Section 4.2, in which equivocating senders are excluded and the influence of consistent false states is bounded by the screening threshold (Proposition 3).

## 3. Preliminaries

This section reviews the estimation, optimization, and blockchain background.

### 3.1. Cooperative UAV Localization and Weighted Least Squares

Cooperative localization combines an absolute position source, such as GNSS, with relative measurements between neighboring UAVs. The absolute measurements anchor the estimates in a common frame, whereas relative measurements couple neighboring estimates. WLS assigns each measurement a weight equal to its inverse variance; for independent Gaussian errors, the WLS estimate is the maximum-likelihood estimate.

### 3.2. Distributed Optimization via ADMM

ADMM converts a coupled problem into local updates linked by consistency constraints. Each UAV updates its own state, edge-associated copies are updated for neighboring pairs, and scaled dual variables accumulate the disagreement between copies and states. For convex problems with a saddle point, the iterates converge to an optimal solution for any ρ>0 [18], also under summable errors [50].

### 3.3. Permissioned Blockchain and Smart Contracts

A blockchain is a distributed, append-only ledger whose records are grouped into blocks. Each block contains the hash of its predecessor; hence, any modification in an earlier block can be detected [27,28,29]. A transaction is a signed record submitted to the blockchain. In a permissioned blockchain, only authorized accounts may submit transactions or seal blocks. A smart contract is a program stored on the blockchain and executed identically by all nodes according to predefined rules [51]. A state digest is a fixed-length cryptographic hash of a state record, and signing the digest binds the sender to the record. A nonce makes the digests of identical states distinct, whereas replays are rejected by the contract, which accepts records only from registered UAV addresses and stores one record per UAV, session, and iteration.

### 3.4. Clique Proof of Authority

Our prototype uses Clique, the proof-of-authority engine of Go Ethereum (Geth) [52,53]. A fixed set of *M* authorized signers takes turns sealing blocks at a configured period. To limit the influence of any single signer, a signer may seal at most one of every ⌊M/2⌋+1 consecutive blocks; competing branches are resolved by preferring the chain sealed by in-turn signers. Clique does not collect a quorum certificate from several validators for each block, and its finality is therefore probabilistic; a block becomes increasingly unlikely to be reverted as later blocks extend it. Its safety relies on fewer than half of the signers being malicious at the consensus layer. We call a record *finalized* when it is in a sealed block and has been read after one confirmation (assumptions in Section 4.2).

## 4. Problem Formulation

This section defines the localization problem, the threat model, the attacks considered in this paper, and the distributed ADMM formulation that these attacks target.

### 4.1. Cooperative Localization Model

Let $U=\{U_{0},U_{1},\ldots ,U_{N-1}\}$ denote the set of *N* UAVs, and let $p_{i}^{★}\in R^{2}$ be the true position of $U_{i}$. The communication structure is the undirected graph G=(U,E), where (i,j)∈E means that $U_{i}$ and $U_{j}$ exchange localization information and measure their relative displacement, and $N_{i}=\left\{j|\left(i,j\right)\in E\right\}$ denotes the neighbor set of $U_{i}$. Each UAV has a noisy absolute prior:

(1) $$g_{i}=p_{i}^{★}+\eta_{i}^{g},\;E\left[\eta_{i}^{g}\right]=0,\;E\left[\eta_{i}^{g}{\left(\eta_{i}^{g}\right)}^{T}\right]=\sigma_{g,i}^{2}I_{2},$$

and for every (i,j)∈E the relative displacement is measured as follows:

(2) $$y_{ij}=p_{i}^{★}-p_{j}^{★}+\eta_{ij}^{y},\;E\left[\eta_{ij}^{y}{\left(\eta_{ij}^{y}\right)}^{T}\right]=\sigma_{y,ij}^{2}I_{2},\;y_{ji}=-y_{ij}.$$

**Figure 1:**
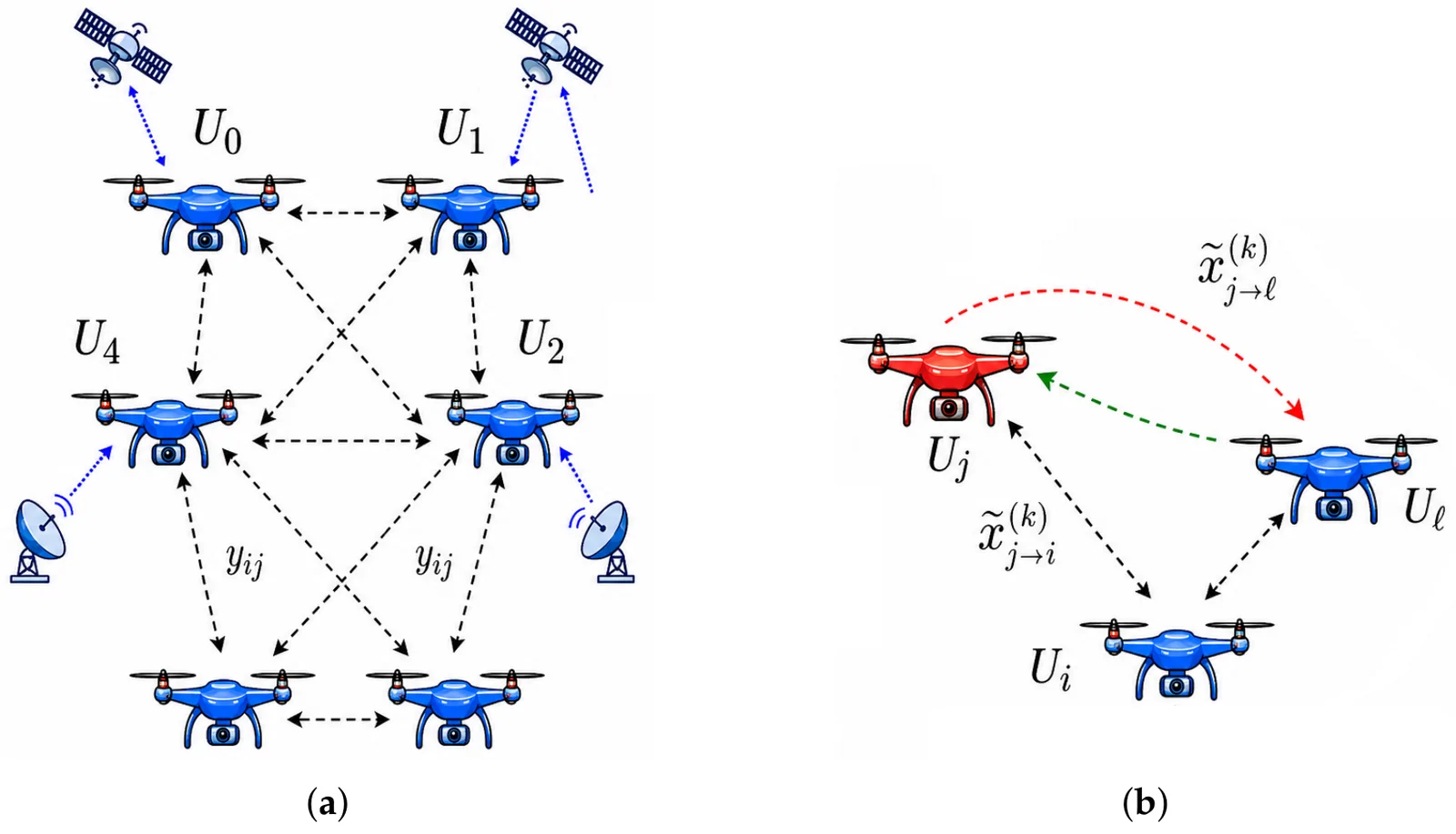
Cooperative UAV localization and receiver-dependent Byzantine manipulation. (**a**) Cooperative UAV localization model, (**b**) Receiver-dependent (equivocating) Byzantine messages.

Let $x_{i}\in R^{2}$ denote the position estimate (state) of $U_{i}$ and X the stacked state. The WLS problem is:

(3) $$\min_{X}J\left(X\right),\;J\left(X\right)=\frac{1}{2}\sum_{i=0}^{N-1}w_{i}∥x_{i}-g_{i}{∥}_{2}^{2}+\frac{1}{2}\sum_{(i,j)\in E,\;i<j}\theta_{ij}{∥\left(x_{i}-x_{j}\right)-y_{ij}∥}_{2}^{2},$$

with $w_{i}=\sigma_{g,i}^{-2}$ and $\theta_{ij}=\sigma_{y,ij}^{-2}$. We consider a static two-dimensional configuration; the implications for moving UAVs are discussed in Section 8. Figure 1 illustrates the cooperative model and the receiver-dependent manipulation of messages; the graph is schematic, and the evaluated topologies are specified in Section 6.

### 4.2. Threat Model and Trust Assumptions

The model above assumes that every UAV reports its state honestly; the threat model relaxes this assumption. Let A⊂U be the set of compromised UAVs, which is unknown to the defense, and H=U∖A the set of honest UAVs.

**Capabilities of a compromised UAV:** A UAV $U_{j}\in A$ controls its own state messages, its blockchain submissions, and its signing key. It may (i) send different states to different neighbors over off-chain channels, (ii) submit several different records to the blockchain for the same iteration, (iii) submit one consistent but false record, (iv) change its falsified values over time, and (v) know the algorithm, noise levels, and threshold and observe its neighbors’ current states before choosing its own, and compromised UAVs may collude.

**Limitations of the attacker:** A compromised UAV cannot forge the signature of an honest UAV, cannot modify records that are already finalized, and cannot alter the absolute priors or relative measurements that honest UAVs obtain from their own sensors. GNSS spoofing of honest UAVs and spoofing of relative measurements are outside the scope of this paper and are addressed by the sensing-level methods in Section 2.

**Blockchain and validators:** The blockchain is a permissioned Clique network with *M* signers (Section 3.4). In our prototype, the six UAV nodes are also the six signers, and a compromised UAV therefore also holds a signer key. We assume that fewer than half of the signers deviate at the consensus layer and that signers do not censor or reorder records; under this assumption, a record that is sealed in a block and read after one confirmation is treated as final. Such a record can still be reverted if a competing block replaces the block that contains it, and for an equivocating sender the replacement could change which of its records is stored first; the honest-majority assumption makes such reorganizations rare and short, and each additional confirmation lowers this risk at the cost of one block period per iteration. Consensus-layer attacks, such as censorship or long-range forks by colluding signers, are out of scope; the compromised UAVs act only at the application layer.

**Information flow:** In the protected protocol, honest UAVs use neighbor states only from finalized blockchain records. Off-chain peer-to-peer messages exist only in the unprotected baseline, in which each receiver uses the states it receives without verification.

**Guarantees:** Within this model, the framework provides three security properties.

- *(S1) Non-equivocation:* All honest UAVs use the same finalized record of a given sender in a given iteration.
- *(S2) Accountability:* A conflicting second record from the same sender and iteration is recorded and flags the sender as inconsistent, which lets honest UAVs exclude it. A conflict is attributed to a sender only on evidence signed by that sender (Section 5.3).
- *(S3) Bounded influence:* After the warm-up, an accepted neighbor state lies within the threshold of the receiver’s prediction, and Proposition 3 bounds the resulting bias.

The framework does *not* guarantee that a single committed state is correct. A compromised UAV can commit a single false state, which the blockchain records like any other state, and the screening accepts it if it lies within the threshold. Our robustness claims are limited to (S1)–(S3), and the adaptive attacker of Section 4.3 quantifies the residual effect.

### 4.3. Attack Models

Within this threat model, we consider four attacks on the exchanged states. Let $T_{j}\subseteq N_{j}$ denote the targeted neighbors of $U_{j}\in A$, and let ${\tilde{x}}_{j\to i}^{\left(k\right)}$ be the state that $U_{i}$ receives at iteration *k*, where $x_{j}^{\left(k\right)}$ is the state the compromised UAV would send if it were honest.

**Byzantine deception (equivocation):** The sender transmits receiver-dependent states:

(4) $${\tilde{x}}_{j\to i}^{\left(k\right)}=x_{j}^{\left(k\right)}+\gamma_{b}\;d_{j\to i},\;i\in T_{j},$$

with magnitude $\gamma_{b}\geq 0$ and unit directions $d_{j\to i}$ that differ across receivers, such that honest neighbors receive conflicting states in the same iteration.

**Byzantine disruption:** The receiver-dependent perturbation switches sign over time:

(5) $${\tilde{x}}_{j\to i}^{\left(k\right)}=x_{j}^{\left(k\right)}+\gamma_{s}\chi^{\left(k\right)}d_{j\to i},\;\chi^{\left(k\right)}={\left(-1\right)}^{\left\lfloor k/L\right\rfloor },$$

where *L* is the number of iterations between sign changes; this attack can produce oscillations or prevent convergence.

**FDI deception:** The sender transmits one consistent false state, ${\tilde{x}}_{j\to i}^{\left(k\right)}=x_{j}^{\left(k\right)}+\gamma_{f}d_{j}$ for all $i\in T_{j}$. Being identical for all receivers, this state cannot be detected as a conflict.

**Behavior under the blockchain:** With the blockchain, an equivocating attacker either submits its receiver-dependent values as several records (the first is finalized and the others are flagged, property (S2)) or commits a *single* false value. The single-value variants of the Byzantine and disruption attacks are therefore evaluated separately. They are the rational response to the blockchain and act like FDI with the original offsets.

**Adaptive attacker:** An adaptive attacker knows the screening rule of Section 5.4, including its threshold, and adapts its false state in every iteration to evade the screening. It commits a value at $\gamma_{a}$ times the smallest threshold from its targets’ mean prediction:

(6) $${\tilde{x}}_{j}^{\left(k\right)}=\frac{1}{|T_{j}|}\sum_{i\in T_{j}}\left({\bar{x}}_{i}^{\left(k\right)}-y_{ij}\right)+\gamma_{a}\;\min_{i\in T_{j}}\tau_{ij}\;d_{j},\;0<\gamma_{a},$$

where ${\bar{x}}_{i}^{\left(k\right)}$ are the current states of the targets. For $\gamma_{a}<1$, this value lies inside the screening region of every target whose prediction is close to the mean, but not necessarily of all targets, and not every adaptive message is accepted. This corresponds to the construction ${\bar{x}}_{j}={\bar{x}}_{i}-y_{ij}+\delta$ with ${∥\delta ∥}_{2}\leq \tau_{ij}$, re-centered on the current states of the targets in every iteration.

### 4.4. Distributed ADMM Reformulation

These attacks target the messages of a distributed solver of (3), which we obtain by the following ADMM reformulation. For each undirected edge, let $x_{ij}$ and $x_{ji}$ denote edge-associated copies of $x_{i}$ and $x_{j}$. Problem (3) is equivalent to:

(7) $$\min_{\left\{x_{i}\right\},\left\{x_{ij},x_{ji}\right\}}\;\sum_{i}\phi_{i}\left(x_{i}\right)+\sum_{(i,j)\in E,\;i<j}\psi_{ij}\left(x_{ij},x_{ji}\right)\;s.t.\;x_{ij}=x_{i},\;x_{ji}=x_{j},$$

with $\phi_{i}\left(x_{i}\right)=\frac{w_{i}}{2}{∥x_{i}-g_{i}∥}_{2}^{2}$ and $\psi_{ij}\left(x_{ij},x_{ji}\right)=\frac{\theta_{ij}}{2}{∥\left(x_{ij}-x_{ji}\right)-y_{ij}∥}_{2}^{2}$. Let ρ>0 be the penalty parameter and $u_{ij}$ the scaled dual variable of the constraint $x_{ij}=x_{i}$.

## 5. Proposed Secure Distributed Optimization Framework

To counter the attacks of Section 4.3 within the threat model of Section 4.2, this section presents the proposed framework and analyzes its properties.

### 5.1. Framework Overview

**Figure 2:**
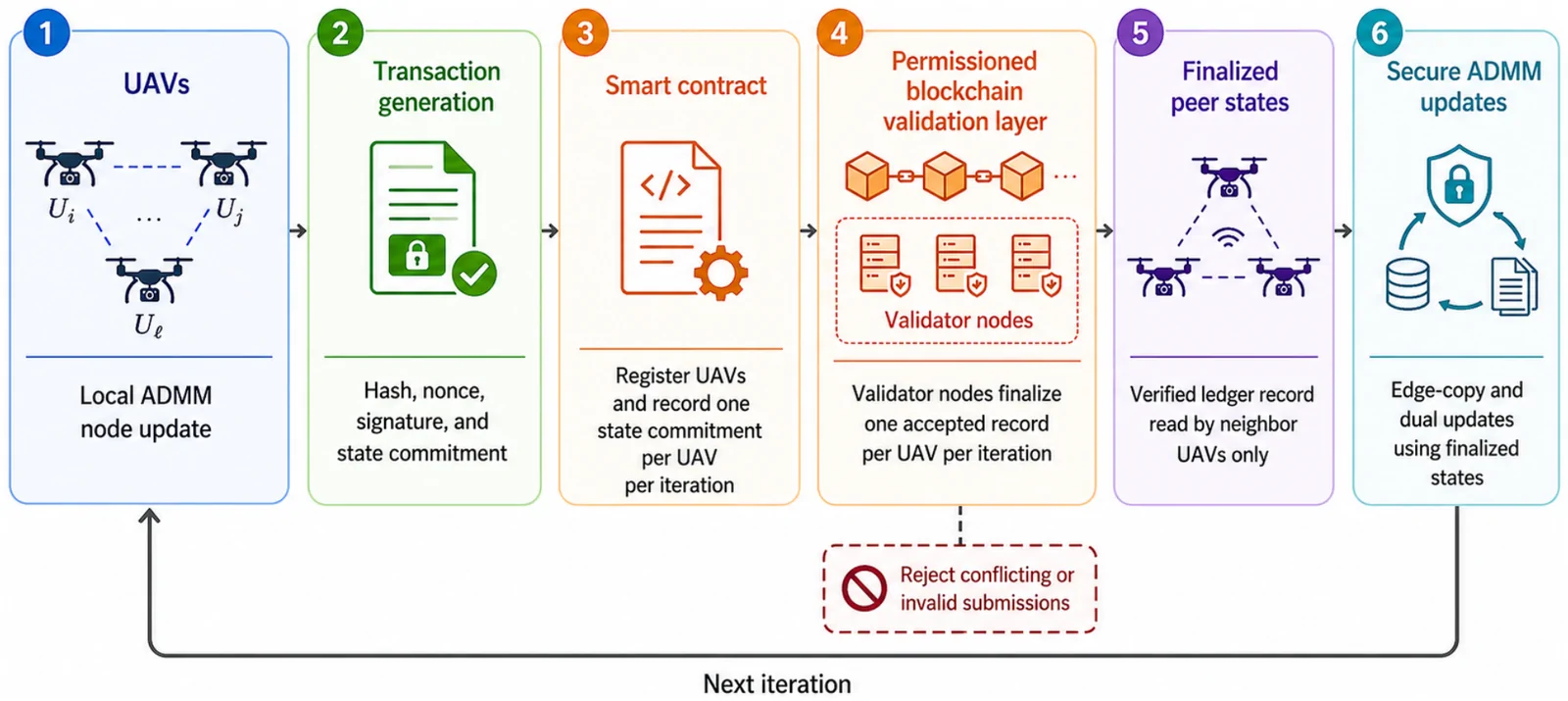
Workflow of the proposed secure distributed localization framework, from the local ADMM update to the blockchain-based acceptance of state records and the secure edge updates.

The proposed defense combines two mechanisms with complementary roles, the blockchain and the relative-measurement screening (Figure 2). The blockchain accepts one signed record per UAV and iteration and flags conflicting records, which provides (S1) and (S2). Each honest UAV then compares every finalized neighbor state with the state predicted from its own relative measurement and replaces states that are inconsistent with the measured geometry, which provides (S3). Each UAV performs the ADMM edge updates on its own copy of every incident edge, and no UAV therefore relies on the screening decisions of a neighbor. In Figure 2, the state commitment is the digest of Equation (9), and the validator nodes are the Clique signers. Each iteration consists of a local update, the acceptance of the resulting records by the blockchain, the screening of the accepted states, and the edge updates, which Section 5.2, Section 5.3, Section 5.4 and Section 5.5 describe, respectively.

### 5.2. Local Update and Signed Record Generation

At iteration k+1, UAV $U_{i}$ updates its state using only its own edge copies and duals as follows:

(8) $$x_{i}^{\left(k+1\right)}=\arg\min_{x_{i}}\left\{\phi_{i}\left(x_{i}\right)+\frac{\rho }{2}\sum_{j\in N_{i}}{∥x_{ij}^{\left(k\right)}-x_{i}+u_{ij}^{\left(k\right)}∥}_{2}^{2}\right\}=\frac{w_{i}g_{i}+\rho \sum_{j\in N_{i}}\left(x_{ij}^{\left(k\right)}+u_{ij}^{\left(k\right)}\right)}{w_{i}+\rho \left|N_{i}\right|}.$$

It then forms the state record $m_{i}^{\left(k+1\right)}$ containing $x_{i}^{\left(k+1\right)}$, and computes the digest and signature:

(9) $$c_{i}^{\left(k+1\right)}=H\left(i\;∥\;\zeta \;∥\;k+1\;∥\;m_{i}^{\left(k+1\right)}\;∥\;\omega_{i}^{\left(k+1\right)}\right),\;\xi_{i}^{\left(k+1\right)}=S_{\nu_{i}}\left(c_{i}^{\left(k+1\right)}\right),$$

where ζ is the session identifier, $\omega_{i}^{\left(k+1\right)}$ a one-time random value, H(·) a cryptographic hash function, and $S_{\nu_{i}}$ the signature with the private key $\nu_{i}$. The record $m_{i}^{\left(k+1\right)}$ consists of the two coordinates of $x_{i}^{\left(k+1\right)}$ in a fixed-point format with a resolution of $10^{-6}$ m, $\omega_{i}^{\left(k+1\right)}$ has 32 bytes, and *H* is Keccak-256 applied to the ABI encoding of the fields in the order of Equation (9); the UAV client and the smart contract use this same construction. The UAV submits $z_{i}^{\left(k+1\right)}=\left(i,\zeta ,k+1,m_{i}^{\left(k+1\right)},\omega_{i}^{\left(k+1\right)},c_{i}^{\left(k+1\right)},\xi_{i}^{\left(k+1\right)}\right)$ to the smart contract as a transaction, whose signature with $\nu_{i}$ serves as $\xi_{i}^{\left(k+1\right)}$. The contract recomputes $c_{i}^{\left(k+1\right)}$ from the submitted coordinates and $\omega_{i}^{\left(k+1\right)}$ and rejects the record if the result differs from the submitted commitment; every stored state is therefore bound to its commitment, which anyone can recompute from the stored record. A UAV that also sends its state off chain attaches its signature of $c_{i}^{\left(k+1\right)}$, which additionally covers the contract address and the chain identifier and serves as evidence in Section 5.3.

### 5.3. Blockchain Acceptance and Exclusion of Equivocating Senders

When the records arrive, the smart contract enforces the acceptance rule. For each tuple (i,ζ,k+1), the contract stores the first valid record and emits a commitment event. A later record with the same tuple and different coordinates is not stored; instead, the contract records the conflict, emits an equivocation event, and marks the sender as inconsistent for that iteration, whereas a resubmission of the same coordinates is ignored. A receiver that obtained a state of the sender off chain can report it, and the contract marks the sender as inconsistent if this state differs from the stored one. The contract accepts such a report only if it carries the sender’s signature on the commitment of the reported state for the same session and iteration. Because a compromised UAV cannot forge the signature of an honest UAV (Section 4.2), it cannot cause the exclusion of an honest UAV, and every recorded conflict is supported by a transaction or a signature of the flagged sender. In the protected protocol, neighbor states are read only from the chain; the localization runs therefore use no such reports. Each iteration closes at a common block height $h_{k+1}=h_{0}+\left(k+1\right)\Delta$, where the session start block $h_{0}$ and the period Δ (in blocks) are session parameters, so that every honest UAV derives the same closing block from its view of the canonical chain; in the prototype, the coordinating script instead closed each iteration once all of its records were included, which corresponds to a period long enough for all records. After block $h_{k+1}$ is sealed and confirmed, each UAV reads the finalized states ${\bar{x}}_{j}^{\left(k+1\right)}$ of its neighbors and the consistency flags as of this block; the state of a sender flagged as inconsistent is excluded in that iteration (it is replaced as in Equation (11)). A neighbor without a record up to block $h_{k+1}$, for example because it failed to submit one, is treated in the same way, and records or conflicts included after this block do not affect the iteration but remain on the chain for accountability; a sender that submits its conflict late is thus treated like a single false value, which the screening handles. This treatment of missing records and of records or conflicts included after the closing block is part of the protocol specification but is not implemented in the prototype, whose script closes an iteration only after all records are included; in all runs, every UAV submitted its records in every iteration. Missing submissions, deadline expiry, and late conflicts have therefore been neither implemented nor validated experimentally; Table 2 lists the implemented operations and where each is evaluated. Because all honest UAVs read the same records and flags as of the same canonical block, property (S1) holds regardless of the communication topology. A later reorganization of this block may temporarily break this common view; recovery from such reads is not evaluated, and the convergence results apply once their assumptions hold.

### 5.4. Relative-Measurement Screening

After reading the finalized states, receiver $U_{i}$ predicts the state of neighbor $U_{j}$ from its own state and relative measurement and computes the screening residual:

(10) $${\hat{x}}_{j|i}^{\left(k+1\right)}={\bar{x}}_{i}^{\left(k+1\right)}-y_{ij},\;e_{j|i}^{\left(k+1\right)}={\bar{x}}_{j}^{\left(k+1\right)}-{\hat{x}}_{j|i}^{\left(k+1\right)},\;r_{j|i}^{\left(k+1\right)}={∥e_{j|i}^{\left(k+1\right)}∥}_{2}.$$

For k+1>W, where *W* denotes the length of the warm-up period, the state is flagged when $r_{j|i}^{\left(k+1\right)}>\tau_{ij}$, with $\tau_{ij}=\kappa \;\sigma_{y,ij}$. A flagged or excluded state is replaced by the prediction,

(11) $${\overset{ˇ}{x}}_{j|i}^{\left(k+1\right)}={\hat{x}}_{j|i}^{\left(k+1\right)},$$

and otherwise ${\overset{ˇ}{x}}_{j|i}^{\left(k+1\right)}={\bar{x}}_{j}^{\left(k+1\right)}$. Replacement removes the information carried by the edge in that iteration. We also evaluate a clipping variant, ${\overset{ˇ}{x}}_{j|i}={\hat{x}}_{j|i}+\tau_{ij}e_{j|i}/r_{j|i}$, which retains a bounded part of the flagged state.

**Calibration of the threshold:** The residual contains the estimation errors of both UAVs and not only the relative-measurement noise. If ${\bar{x}}_{i}=g_{i}$ and ${\bar{x}}_{j}=g_{j}$ (the initialization), then $e_{j|i}=\eta_{j}^{g}-\eta_{i}^{g}+\eta_{ij}^{y}$ with covariance $\left(\sigma_{g,i}^{2}+\sigma_{g,j}^{2}+\sigma_{y,ij}^{2}\right)I_{2}$, and a threshold of a few $\sigma_{y,ij}$ rejects a large fraction of honest states; with $\sigma_{g}=\sigma_{y}$ and κ=2 the rejection probability is exp(−2/3)≈51%. Rejected honest edges no longer pull neighboring estimates toward each other, and the rejections can persist. Near the attack-free solution, however, the residual is small, as the following lemma shows.

**Lemma** **1** (False alarms near the attack-free solution)**.** *Let X be the WLS solution of (3) under Gaussian noise. Then, for every edge (i,j), $e_{j|i}=-\left(x_{i}-x_{j}-y_{ij}\right)$ is a zero-mean Gaussian vector with covariance $⪯\sigma_{y,ij}^{2}I_{2}$, and $\mathrm{Pr}\left\{r_{j|i}>\kappa \sigma_{y,ij}\right\}\leq \exp\left(-\kappa^{2}/2\right)$.*

**Proof.**  Write the measurements of (3) as $b=Ap^{★}+\eta$ with noise covariance Σ. The WLS residual $b-A\hat{X}$ has covariance $\Sigma -A{\left(A^{T}\Sigma^{-1}A\right)}^{-1}A^{T}⪯\Sigma$; the block of the relative measurement (i,j) is therefore $⪯\sigma_{y,ij}^{2}I_{2}$. Writing $e_{j|i}=C^{1/2}z$ with $z∼N(0,I_{2})$ and $C⪯\sigma_{y,ij}^{2}I_{2}$ gives $r_{j|i}^{2}\leq \sigma_{y,ij}^{2}{∥z∥}^{2}$, and ${\mathrm{Pr}\{∥z∥}^{2}>\kappa^{2}\}=\exp\left(-\kappa^{2}/2\right)$ for a chi-square variable with two degrees of freedom.    □

Lemma 1 motivates the fixed rule $\tau_{ij}=\kappa \sigma_{y,ij}$ together with a warm-up of *W* iterations, during which the estimates move from the priors toward the WLS solution and the residual distribution approaches that of the lemma. With κ=2.5, the bound gives a nominal false-alarm rate of at most exp(−3.125)≈4.4% per message. A threshold that also accounts for the prior uncertainty, $\tau_{ij}=\sqrt{-2\ln\alpha }\;{\left(\sigma_{y,ij}^{2}+\sigma_{g,i}^{2}+\sigma_{g,j}^{2}\right)}^{1/2}$, is valid from the first iteration but is so large (6.6 m for α=0.01 here) that it accepts the evaluated attacks (Section 7.4).

### 5.5. Symmetric Secure Edge Updates

In a literal implementation of (7), the joint update of $\left(x_{ij},x_{ji}\right)$ for an edge with i<j would be computed with the screening result of one endpoint only. This makes the update depend on the arbitrary UAV indices, and when the lower-index endpoint is compromised it relies on a compromised UAV to perform the screening. We eliminate both problems by letting every UAV maintain its own copy of each incident edge subproblem. For each neighbor $j\in N_{i}$, UAV $U_{i}$ stores its own copy $x_{ij}$ and dual $u_{ij}$, and a local copy $x_{ji}^{\left[i\right]}$ of the neighbor’s edge variable with dual $u_{ji}^{\left[i\right]}$. Using its screened view ${\overset{ˇ}{x}}_{j|i}$, UAV $U_{i}$ solves:

(12) $$\left(x_{ij}^{\left(k+1\right)},x_{ji}^{\left[i](k+1\right)}\right)=\arg\min_{a,b}\left\{\frac{\theta_{ij}}{2}∥\left(a-b\right)-y_{ij}{∥}_{2}^{2}+\frac{\rho }{2}∥a-c_{ij}{∥}_{2}^{2}+\frac{\rho }{2}{∥b-c_{ji}^{\left[i\right]}∥}_{2}^{2}\right\},$$

with $c_{ij}={\bar{x}}_{i}^{\left(k+1\right)}-u_{ij}^{\left(k\right)}$ and $c_{ji}^{\left[i\right]}={\overset{ˇ}{x}}_{j|i}^{\left(k+1\right)}-u_{ji}^{\left[i](k\right)}$. The minimizer has the closed form

(13) $$x_{ij}^{\left(k+1\right)}=c_{ij}+f_{ij}d_{ij},\;x_{ji}^{\left[i](k+1\right)}=c_{ji}^{\left[i\right]}-f_{ij}d_{ij},\;d_{ij}=y_{ij}-c_{ij}+c_{ji}^{\left[i\right]},\;f_{ij}=\frac{\theta_{ij}}{2\theta_{ij}+\rho },$$

and the duals are updated as follows:

(14) $$u_{ij}^{\left(k+1\right)}=u_{ij}^{\left(k\right)}+x_{ij}^{\left(k+1\right)}-{\bar{x}}_{i}^{\left(k+1\right)},\;u_{ji}^{\left[i](k+1\right)}=u_{ji}^{\left[i](k\right)}+x_{ji}^{\left[i](k+1\right)}-{\overset{ˇ}{x}}_{j|i}^{\left(k+1\right)}.$$

Only $x_{ij}$ and $u_{ij}$ enter the node update (8); the local copy of the neighbor’s variable is used only to evaluate (12). Consequently, each UAV computes every quantity that it uses from finalized neighbor states and its own measurements, and it does not depend on any computation performed by a neighbor; Algorithm 1 summarizes the procedure. The iteration stops when the RMS own-copy primal residual $r_{\mathrm{own}}$ (computed from $∥x_{ij}-{\bar{x}}_{i}∥$), the RMS screened primal residual $r_{\mathrm{scr}}$ (computed from $∥x_{ji}^{\left[i\right]}-{\overset{ˇ}{x}}_{j|i}∥$), and the RMS dual residual $s=\rho \cdot \mathrm{RMS}∥x_{ij}^{\left(k+1\right)}-x_{ij}^{\left(k\right)}∥$, computed over the honest UAVs, are below $\epsilon_{p}$ and $\epsilon_{d}$. This evaluation criterion uses the honest set, which is unknown during operation; a deployed network instead uses a shared iteration budget, during which every UAV keeps updating and submitting its state, so that no neighbor is treated as missing. We also report the original primal residual $r_{\mathrm{orig}}$, computed from $∥x_{ji}^{\left[i\right]}-{\bar{x}}_{j}∥$, which measures consistency with the finalized states and does not vanish when a state is replaced.
**Algorithm 1** Secure blockchain-assisted ADMM (operations of one honest UAV $U_{i}$)**Require:** Session ζ, neighbors $N_{i}$, prior $g_{i}$, measurements $\left\{y_{ij}\right\}$
**Require:** Penalty ρ, factor κ, warm-up *W*, tolerances $\epsilon_{p},\epsilon_{d}$  1: Initialize $x_{i}^{\left(0\right)}=g_{i}$ and, for all $j\in N_{i}$, set $x_{ij}^{\left(0\right)}=g_{i}$, $x_{ji}^{\left[i](0\right)}=g_{j}$, and zero duals  2: **for** 
k=0,1,2,…
 **do**  3:      Compute $x_{i}^{\left(k+1\right)}$ with (8); form, sign, and submit the record $z_{i}^{\left(k+1\right)}$  4:      Wait until the closing block $h_{k+1}$ is sealed and confirmed; read ${\bar{x}}_{j}^{\left(k+1\right)}$ and the consistency flags of all $j\in N_{i}$ as of this block  5:      **for all** $j\in N_{i}$ **do**  6:            Compute the prediction ${\hat{x}}_{j|i}={\bar{x}}_{i}^{\left(k+1\right)}-y_{ij}$  7:            **if** $U_{j}$ has no record or is flagged as inconsistent **then**  8:                   ${\overset{ˇ}{x}}_{j|i}\leftarrow {\hat{x}}_{j|i}$▹ exclusion  9:            **else if** k+1>W and $r_{j|i}>\kappa \sigma_{y,ij}$, with $r_{j|i}$ from (10) **then**10:                   ${\overset{ˇ}{x}}_{j|i}\leftarrow {\hat{x}}_{j|i}$▹ replacement11:            **else** 12:                   ${\overset{ˇ}{x}}_{j|i}\leftarrow {\bar{x}}_{j}^{\left(k+1\right)}$ 13:
            **end if** 14:
            Update $x_{ij}$, $x_{ji}^{\left[i\right]}$ with (13) and $u_{ij}$, $u_{ji}^{\left[i\right]}$ with (14) 15:      **end for** 16:      **if** the stopping rule of Section 5.5 is met **then stop** 17:      **end if** 18: **end for**

**Example** **1.** *Consider two UAVs with states (0,0) and (10,0), y=(0,0), τ=1 m, and zero duals. With the one-sided edge update, the edge copies become ((0,0),(0,0)) under one labeling and ((10,0),(10,0)) when the indices are exchanged. Because the residual of 10 m exceeds τ, each endpoint flags the state of the other. With the symmetric update (12), each UAV therefore replaces the state of the other by its own prediction and keeps its own copy at its own state, (0,0) and (10,0), under both labelings.*

### 5.6. Theoretical Properties

We analyze the secure iteration in three situations, namely without rejections, under stabilized screening decisions, and at fixed points with accepted compromised states.

**Proposition** **1** (Equivalence and label invariance)**.** *(a) If no state is flagged or excluded, i.e., ${\overset{ˇ}{x}}_{j|i}={\bar{x}}_{j}$ for all honest receivers and iterations, and no UAV is compromised, then $x_{ji}^{\left[i](k\right)}=x_{ji}^{\left(k\right)}$ and $u_{ji}^{\left[i](k\right)}=u_{ji}^{\left(k\right)}$ for all k, and the iteration coincides with standard ADMM applied to (7); it converges to the WLS solution of (3) for every ρ>0. (b) For any consistent permutation of the indices in priors, measurements, and the compromised set, the iterates are permuted accordingly.*

**Proof.**  (a) The local copies are initialized identically at both endpoints. Suppose that the copies held by $U_{i}$ equal the variables held by $U_{j}$ at iteration *k*. With $y_{ji}=-y_{ij}$, problem (12) solved by $U_{j}$ is the mirror image of the one solved by $U_{i}$ with identical centers; both endpoints therefore obtain the same minimizer, and (14) preserves the equality. By induction, the iteration is the standard two-block ADMM for the convex problem (7), which converges for any ρ>0 [18]. (b) Every quantity of $U_{i}$ is indexed by (i,j) and is defined regardless of the order of *i* and *j*; relabeling thus permutes the computations.    □

Numerically, the attack-free iteration reproduces the centralized WLS solution to within $6.4\times 10^{-10}$ m for six UAVs and $1.9\times 10^{-6}$ m for 1000 UAVs, and random relabeling changes the estimates by at most $1.1\times 10^{-13}$ m under attack.

**Proposition** **2** (Convergence under stabilized screening decisions)**.** *Suppose that, from some iteration K on, (i) the decision of every honest UAV for each neighbor (accept, replace, or exclude) no longer changes, (ii) honest UAVs accept each other mutually, and (iii) every state sent by a compromised UAV to an honest UAV is replaced or excluded. Then, the honest estimates converge to the WLS solution of the honest subnetwork restricted to the accepted edges; if no honest state is rejected, this is the ideal WLS solution, i.e., the WLS solution with all links of compromised UAVs removed, which could be computed only if the compromised UAVs were known.*

**Proof sketch.**  For a replaced edge, ${\overset{ˇ}{x}}_{j|i}={\bar{x}}_{i}-y_{ij}$ depends only on $U_{i}$; substituting into (13) and (14) gives $u_{ij}-u_{ji}^{\left[i\right]}\leftarrow 2f_{ij}\left(u_{ij}-u_{ji}^{\left[i\right]}\right)$ with $2f_{ij}<1$; hence, the replaced edge acts as a zero-cost term with a copy of $x_{i}$ and carries no input from $U_{j}$. For a mutually accepted honest edge, let $e_{1}=u_{ji}^{\left[i\right]}-u_{ji}$ and $e_{2}=u_{ij}^{\left[j\right]}-u_{ij}$ be the mismatches between the copies held by the two endpoints. A direct computation from (13) and (14) gives $e_{1}^{\left(k+1\right)}=e_{2}^{\left(k+1\right)}=f_{ij}\left(e_{1}^{\left(k\right)}+e_{2}^{\left(k\right)}\right)$, and the mismatch therefore decays geometrically with ratio $2f_{ij}=2\theta_{ij}/\left(2\theta_{ij}+\rho \right)<1$. The honest iteration is therefore standard ADMM for the WLS problem of the honest subnetwork with accepted edges, perturbed by summable errors, and converges [18,50].    □

Proposition 2 explains the ideal-WLS accuracy observed when all compromised states are rejected or excluded (Section 7). When compromised states are accepted, the estimates can be biased; the next result bounds this bias at any fixed point, for any attacker.

**Proposition** **3** (Bias bound at fixed points)**.** *Consider a fixed point of the secure iteration reached after the warm-up, in replacement mode, at which honest neighbors accept each other mutually. For an honest receiver $U_{i}$, let $C_{i}\subseteq N_{i}\cap A$ be the compromised neighbors whose states are accepted, and write each accepted state as ${\bar{x}}_{j}={\bar{x}}_{i}-y_{ij}+\delta_{ij}$. By the screening rule, $∥\delta_{ij}{∥}_{2}\leq \tau_{ij}$. Let $x^{o}$ be the WLS solution of the honest subnetwork with its accepted edges. Then, for every i∈H,*

(15) $$∥x_{i}-x_{i}^{o}{∥}_{2}\;\leq \;\frac{1}{\min_{\ell \in H}w_{\ell }}\;\max_{\ell \in H}\sum_{j\in C_{\ell }}\theta_{\ell j}\tau_{\ell j}\;=\;\frac{\sigma_{g}^{2}}{\sigma_{y}^{2}}\;\kappa \;\sigma_{y}\;m_{\max}$$

*where the equality holds for uniform noise levels and $m_{\max}=\max_{\ell }\left|C_{\ell }\right|$. In particular, if all compromised states are rejected at the fixed point, the honest estimates equal $x^{o}$.*

**Proof.**  At a fixed point, $x_{ij}={\bar{x}}_{i}$ and $x_{ji}^{\left[i\right]}={\overset{ˇ}{x}}_{j|i}$. The optimality condition of (12) gives $\rho u_{ij}=-\theta_{ij}\left(x_{i}-{\overset{ˇ}{x}}_{j|i}-y_{ij}\right)$, and (8) gives $w_{i}\left(x_{i}-g_{i}\right)=\rho \sum_{j}u_{ij}$. Hence every honest UAV satisfies:

(16) $$w_{i}\left(x_{i}-g_{i}\right)+\sum_{j\in N_{i}}\theta_{ij}\left(x_{i}-{\overset{ˇ}{x}}_{j|i}-y_{ij}\right)=0.$$

A replaced edge contributes zero, an accepted honest edge contributes $\theta_{ij}\left(x_{i}-x_{j}-y_{ij}\right)$, and an accepted compromised edge contributes $-\theta_{ij}\delta_{ij}$. Stacking (16) over H and subtracting the optimality condition of $x^{o}$ yields $\left(W+L\right)\left(x-x^{o}\right)=F$, where $W=\mathrm{diag}(w_{\ell })$, L is the weighted Laplacian of the accepted honest edges (applied coordinate-wise), and $F_{\ell }=\sum_{j\in C_{\ell }}\theta_{\ell j}\delta_{\ell j}$. The matrix M=W+L has positive diagonal entries, non-positive off-diagonal entries, and is strictly diagonally dominant; it is therefore a nonsingular M-matrix with $M^{-1}\geq 0$ entrywise [54]. Since L1=0, $M^{-1}W1=1$ and hence $\sum_{\ell }{\left[M^{-1}\right]}_{i\ell }\leq 1/\min_{\ell }w_{\ell }$. Therefore $∥x_{i}-x_{i}^{o}{∥}_{2}\leq \sum_{\ell }{\left[M^{-1}\right]}_{i\ell }∥F_{\ell }{∥}_{2}\leq \max_{\ell }{∥F_{\ell }∥}_{2}/\min_{\ell }w_{\ell }$, and $∥F_{\ell }{∥}_{2}\leq \sum_{j\in C_{\ell }}\theta_{\ell j}\tau_{\ell j}$.    □

The bound is a worst-case guarantee that holds for any attacker, including attackers that re-center on the receivers’ states. It shows that the residual bias scales with the threshold $\kappa \sigma_{y}$, with the number of accepted compromised neighbors, and with the ratio $\sigma_{g}^{2}/\sigma_{y}^{2}$, which describes how weakly the absolute priors anchor the network relative to the relative measurements. In our setting ($\sigma_{g}=1.5$ m, $\sigma_{y}=0.5$ m, κ=2.5) the bound is $11.25\;m_{\max}$ m; under the adaptive attacker, the largest deviation of an honest estimate from the ideal WLS solution is 6.3–10.7 m on average over the runs, whereas the bound with $m_{\max}$ replaced by the largest number of compromised neighbors of an honest UAV gives 55–89 m. Because some honest states are also rejected at the end of these runs, $x^{o}$ differs from the ideal WLS solution, and the two quantities are a contextual comparison rather than a direct measure of the tightness of the bound. The bound explains why a smaller κ and better absolute priors reduce them. The bound concerns the deviation from $x^{o}$ rather than the localization error itself and applies only if a fixed point is reached; we do not claim convergence when accepted compromised values vary over time. During changing screening decisions, we have not established that the iteration minimizes a single fixed objective; Equation (16) characterizes its fixed points. the only exception, at the extreme noise ratio $\sigma_{g}/\sigma_{y}=12$, is reported in Section 7.6.

### 5.7. Smart-Contract Operations

The acceptance rule of Section 5.3 is implemented by the smart-contract operations in Table 2, which also lists their gas consumption and where each operation is evaluated.

**Table 2:** Smart-contract operations and their gas consumption. Gas is measured for the current contract on an Ethereum virtual machine with the gas schedule of the prototype network (Section 7.2).

Operation	Purpose	Gas
UAV registration	Maps each authorized UAV address to one UAV index before localization starts (owner only).	–
State-record submission	Recomputes the commitment from the submitted coordinates and nonce, rejects the record if it differs from the submitted commitment, and otherwise stores the first record of a UAV for a session and iteration and emits a commitment event.	256,325
Conflicting record	A record with different coordinates for the same UAV, session, and iteration is not stored; the conflict is recorded, an equivocation event is emitted, and the sender is flagged.	85,679
Resubmission of the same state	A record with the same coordinates, with the same or a new nonce, is ignored and a duplicate event is emitted.	39,323
Receiver report	Records a state of another UAV obtained off chain; accepted only with that UAV’s signature on its commitment, and a signed state that differs from the stored one flags that UAV.	183,018 211,440
Consistency query	Returns whether a conflicting state has been attributed to a UAV in an iteration; used by honest UAVs to exclude equivocating senders.	view
State query	Returns the stored state, nonce, and commitment of a UAV for a session and iteration.	view

## 6. Experimental Setup

This section describes the implementation, the levels at which the proposed defense is evaluated, and the scenarios, attacks, and baselines.

### 6.1. Implementation and Blockchain Prototype

The localization solver is implemented in Python using NumPy and SciPy. The released simulation requirements specify NumPy (version 2.4.4) and SciPy (version 1.17.1), while the figures and tables were reproduced using Python (version 3.12.3). The blockchain-integrated prototype runs six nodes of Geth (version 1.13.14-stable) on a private network using the Clique proof-of-authority consensus engine. Each node holds one UAV account, and the six accounts serve as the Clique signers. The smart contract is compiled using Solidity (version 0.8.0, EVM target istanbul) and deployed from its compiled artifact [55]. The blockchain-integrated solver uses Python (version 3.10.0) and Web3.py (version 8.0.0) [56]. In each iteration, UAVs sign and submit their records and read the confirmed records and consistency flags of their neighbors. The prototype runs on a laptop equipped with an Intel Core i9 processor (2.2 GHz base frequency) and 32 GB of RAM. Table 3 summarizes the experimental parameters and blockchain configuration.

**Evaluation levels:** The evaluation comprises blockchain-integrated runs, a blockchain load test, and numerical simulations. In the *blockchain-integrated runs* of the six-UAV network, every state record is signed, submitted to the Clique network, and read back from it (Section 7.2). In the *blockchain load test*, up to 1000 UAV accounts submit records to the same network in one iteration to measure latency and throughput, in the default and in an optimized configuration (Section 7.7). In the *numerical simulations* of the six- and 1000-UAV networks, the same ADMM and screening are executed with the blockchain replaced by a software model of its acceptance rule, which accepts one record per UAV and iteration and flags conflicting records. The blockchain-integrated runs validate this model, because they reproduce the simulated iteration counts and estimates to within 1.7 µm. All 1000-UAV localization results are therefore simulation results; the cost of the blockchain at this scale is measured separately by the load test. In all cases the blockchain is maintained by the same six validator nodes, and UAVs submit and read records as clients; the number of validators therefore does not grow with the number of UAVs, and in the six-UAV prototype, each UAV also runs one validator node.

### 6.2. Scenarios, Attacks, and Defenses

**Six-UAV network:** The UAVs are placed around six nominal positions with Gaussian jitter (standard deviation 0.8 m), and UAVs within 14 m communicate, which yields the seven edges (0,2), (0,3), (1,2), (2,3), (2,4), (3,4), and (4,5). UAVs $U_{1}$ and $U_{4}$ are compromised and target all their neighbors, i.e., $T_{1}=\left\{2\right\}$ and $T_{4}=\left\{2,3,5\right\}$. This network is a stress test, because one third of the UAVs are compromised and $U_{5}$ is connected only to $U_{4}$.

**1000-UAV network (simulation):** The UAVs are placed uniformly in a 250×250 m^2^ area and connected by the symmetrized 8-nearest-neighbor graph (4665 edges on average). The compromised sets of 100, 200, and 300 UAVs are drawn uniformly at random, and each compromised UAV targets all its neighbors.

**Defenses:** The proposed defense (blockchain + screening, Section 5) is labeled *Proposed* in all tables and figures. We compare it with *no defense* (receivers use the received states); *blockchain only* (states are read from the blockchain and equivocating senders are excluded, without screening); *screening only* (screening of peer-to-peer messages without the blockchain); *signed exchange only* (signed peer-to-peer messages whose digests are forwarded to common neighbors, such that equivocation is detected only when two receivers share a neighbor; no blockchain is used); *signed exchange + screening*; and a *W-MSR baseline* [37], i.e., a W-MSR-inspired filter within the ADMM, in which each UAV discards, per coordinate, up to F=1 neighbor-implied positions above and below its own state and replaces a discarded value by the prediction of Equation (11), so that W-MSR enters the same edge update as the screening. W-MSR with F=1 assumes at most one compromised neighbor per honest UAV, which is violated for 24, 58, and 81% of the honest UAVs with 100, 200, and 300 compromised UAVs and for $U_{2}$ in the six-UAV network; it therefore serves as an adapted heuristic comparator rather than a baseline with resilience guarantees, and Section 7.3 adds a comparison under a placement satisfying this local fault-count condition. The configurations *blockchain only* and *screening only* isolate the two components of the proposed defense, and all configurations use the same ADMM. Table rows are attacks, including the attack-free case, and columns are defenses.

**Statistical protocol:** Each large-scale configuration is run ten times with independent positions, noise realizations, and compromised sets (seeds 7–16). The six-UAV Monte Carlo study uses ten noise and geometry realizations, with the compromised pair either fixed ($U_{1}$, $U_{4}$) or placed at random. We report means and 95% confidence intervals based on the *t*-distribution. Runs that do not meet the stopping criterion within 800 iterations are marked with ^†^ in the tables; for these runs, the reported RMSE is the mean over the last 100 iterations, because the value at the final iteration of a non-convergent run depends on the chosen iteration.

## 7. Results

The results proceed from the six-UAV network, evaluated in simulation and on the blockchain prototype, to the 1000-UAV network, the calibration of the screening, the adaptive attacker, the sensitivity to noise and initialization, and the blockchain cost.

### 7.1. Six-UAV Network

**Figure 3:**
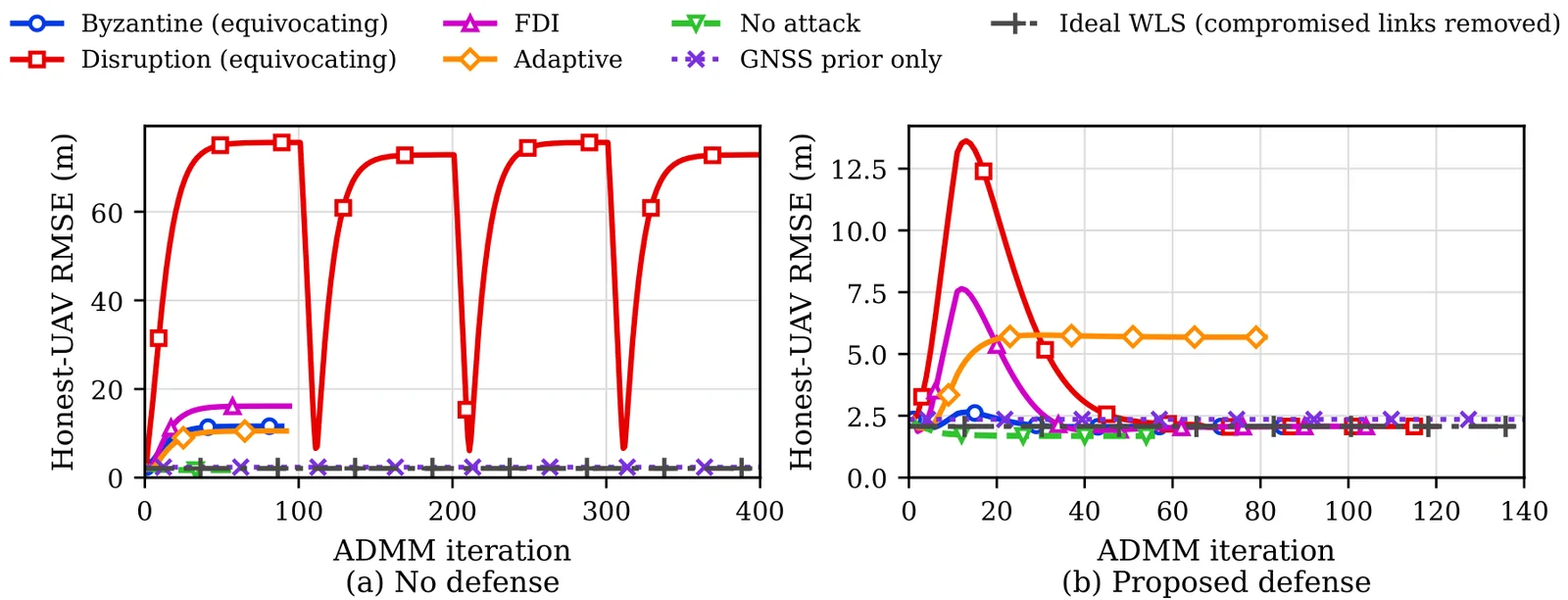
Convergence of the honest-UAV RMSE in the simulated six-UAV network (**a**) without defense and (**b**) with the proposed defense.

**Figure 4:**
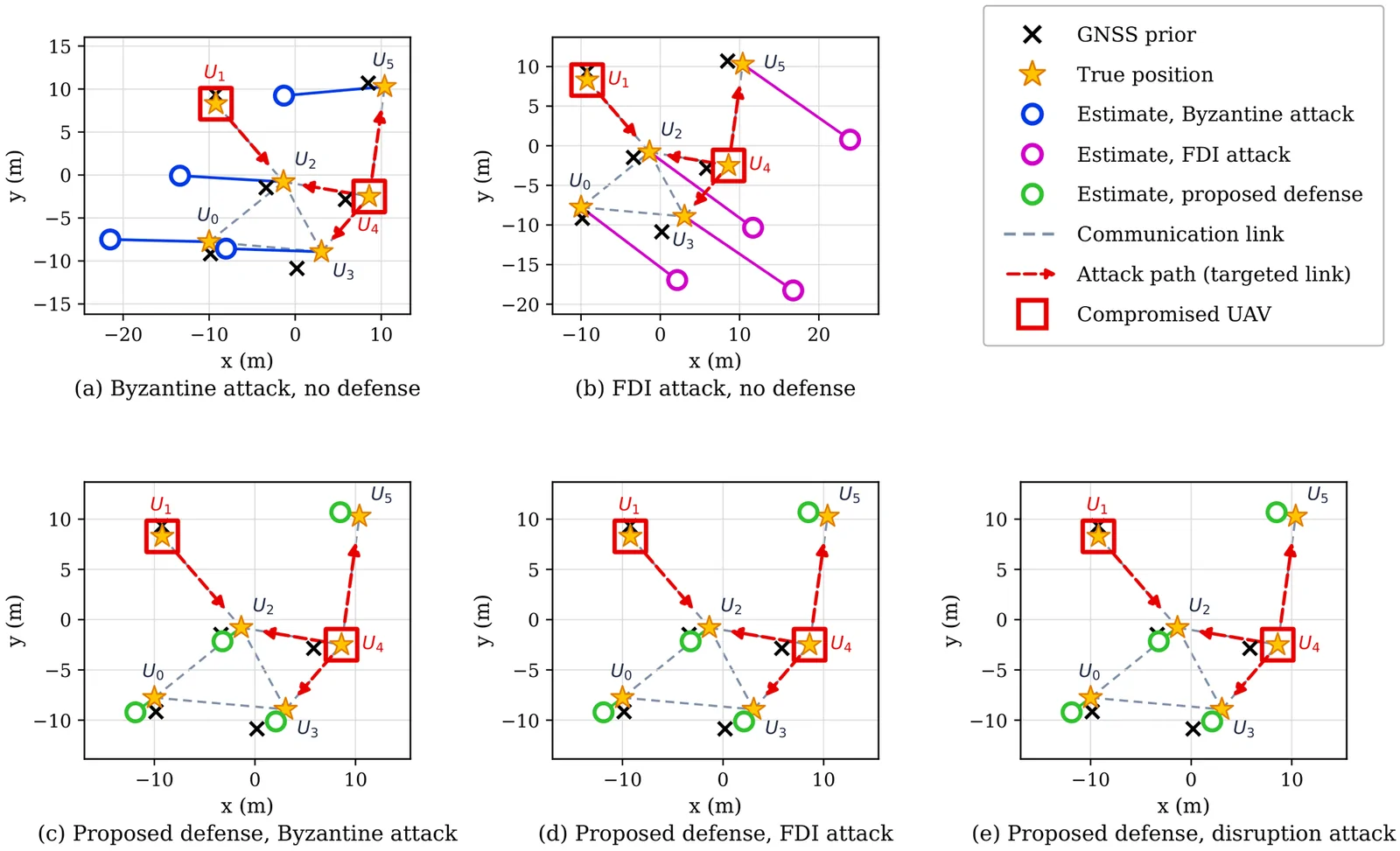
Final position estimates in the simulated six-UAV network under (**a**) Byzantine and (**b**) FDI attacks without defense, and under the proposed defense against (**c**) Byzantine, (**d**) FDI, and (**e**) disruption attacks.

**Figure 5:**
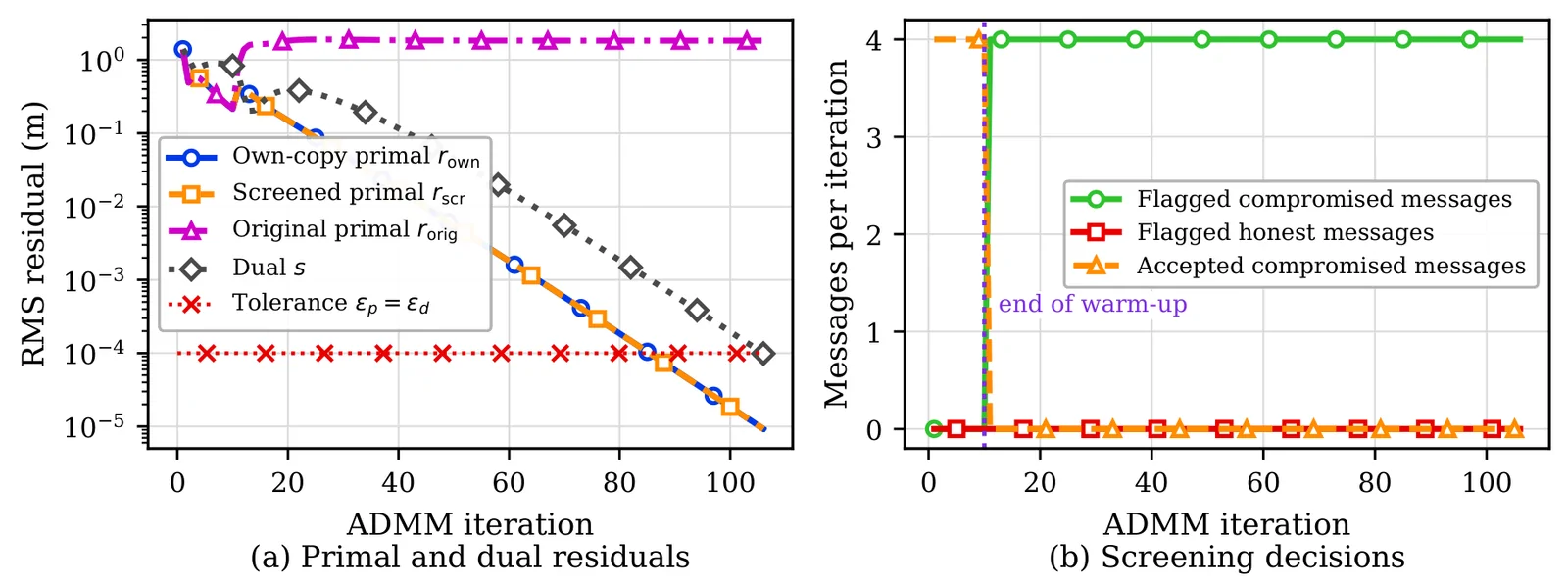
Convergence diagnostics of the proposed defense in the simulated six-UAV network under FDI, showing (**a**) the primal and dual residuals and (**b**) the screening decisions per iteration.

Figure 3, Figure 4 and Figure 5 and Table 4 report simulations of the six-UAV network, which the blockchain-integrated runs of Section 7.2 reproduced. Figure 3 shows the honest-UAV RMSE. Without defense, all attacks drive the estimates away from the true positions. Byzantine deception, FDI, and the adaptive attacker raise the RMSE from 1.68 m to 11.6 m, 16.1 m, and 10.5 m, and disruption produces oscillations between roughly 6 and 76 m that never satisfy the stopping criterion. The errors exceed the injected offsets (1.1–2.8 m) because the false states shift the honest estimates, the compromised UAVs compute their own estimates from these shifted neighbors, and the offset is added again in the next iteration. The bias therefore accumulates until the absolute priors balance it. With the proposed defense, all secured runs converge, and for Byzantine deception, disruption, and FDI the RMSE reaches 2.07 m, which equals the RMSE of the ideal WLS solution that discards all edges of $U_{1}$ and $U_{4}$; this is the outcome predicted by Proposition 2 when every compromised state is excluded or replaced. Under the adaptive attacker, a residual RMSE of 5.68 m remains.

Figure 4 shows the final estimates; the red arrows mark the targeted links, and the undefended disruption case is omitted because it does not converge (Figure 3a). Without defense, the honest UAVs near $U_{1}$ and $U_{4}$ are shifted by several meters in the attack direction (Figure 4a,b). With the defense (Figure 4c–e), the estimates of $U_{0}$, $U_{2}$, and $U_{3}$ are close to their true positions, and $U_{5}$, whose only neighbor is compromised, falls back to its GNSS prior. The three defended maps are nearly identical (differences below 2 mm), as Proposition 2 predicts when every compromised state is excluded or replaced.

Figure 5 reports (a) the primal and dual residuals and (b) the screening decisions per iteration for FDI. The own-copy and screened primal residuals and the dual residual decrease geometrically to the tolerance, whereas the original primal residual stays at the size of the injected offset, because the replaced compromised states are not consistent with the finalized records. This separates the convergence of the optimization variables from the stabilization of the RMSE. During the warm-up, no state is flagged; after the warm-up, all four compromised messages per iteration are flagged and no honest message is rejected.

Table 4 summarizes ten independent realizations. For the fixed compromised pair, the proposed defense reaches the ideal-WLS accuracy under Byzantine deception (1.50 m) and remains within 0.5 m of it under disruption and FDI. Under the single-value Byzantine attack, the result (2.13 m) equals the prior-only accuracy, because the network is small and the screening removes most of the cooperative information around the compromised pair. In this small network, the W-MSR baseline is more accurate than the proposed defense under the single-value Byzantine, disruption, and adaptive attacks (for example 1.84 versus 5.37 m under the adaptive attacker) and less accurate under FDI (2.78 versus 1.72 m); however, depending on the configuration, up to half of its runs do not satisfy the convergence criterion, which is consistent with a trimmed neighbor set that changes from iteration to iteration. These results confirm that the six-UAV network is a stress case; the main conclusions are therefore drawn from the large network.

### 7.2. Blockchain-Integrated Runs

To verify that the simulations reflect the behavior of the blockchain, we ran eight attack and defense configurations of the six-UAV network end to end on the Clique prototype, with the compromised UAVs committing their false states on the chain. Table 5 shows that every on-chain run reproduces the iteration count and RMSE of the simulation; the largest deviation of any estimate is 1.7 µm and stems from the fixed-point encoding. This agreement validates the software model of the blockchain used in the large-scale simulations. In the equivocating Byzantine and disruption runs, $U_{4}$ submits three different records per iteration (one per target), and the contract flags exactly two conflicts per iteration (190 and 240 equivocation events); $U_{1}$ has a single neighbor and cannot equivocate.

In a separate test, one UAV submitted a first record, a conflicting record, and an identical copy of the first record for the same iteration. The first record was finalized; the conflicting record was not stored, triggered an equivocation event, and set the sender’s consistency flag to false; the identical copy was ignored.

**Verification of the record handling:** The runs of Table 5 used a first version of the contract, which did not authenticate receiver reports of conflicting commitments, did not compare the coordinates of a resubmitted record or check them against its commitment, and concatenated the fields of Equation (9) in a different order than the client. The current contract implements Section 5.2 and Section 5.3 as described, which 20 targeted tests on an in-memory Ethereum virtual machine confirm. Reports that were unsigned, signed with the reporter’s own key, signed by the honest UAV for another iteration or another contract deployment, or genuinely signed but with altered coordinates were all rejected, and the honest UAV stayed consistent, also when $U_{1}$ and $U_{4}$ reported fabricated states of their honest neighbors in the six-UAV network; a report carrying the sender’s signature on a state different from its stored one flagged the sender. A record whose commitment did not match its coordinates was rejected, a record whose coordinates differed by 1 µm was flagged as a conflict, and a resubmission of the same coordinates was ignored. The commitments computed by the client, by the contract, and by an independent re-implementation agreed for 1000 random inputs and for all stored records. Re-running the eight configurations of Table 5 through the current contract on the same virtual machine reproduces their iteration counts, RMSE trajectories, and per-iteration numbers of accepted records, equivocation events, and flagged messages exactly, because these runs contain neither reports nor records with mismatched coordinates. The timing in Table 5 and the load test in Section 7.7 were measured with the first version, whose state record needed 278,671 instead of 256,325 gas; for that version, the virtual machine reproduces the gas measured on the prototype to within 24 gas.

On average, an on-chain iteration takes 1.97 s, of which 1.92 s elapse between the submission of the records and their inclusion in a sealed block (including 0.04 s for signing and submission), 0.05 s are spent reading the finalized records, and 0.27 ms are spent on the local ADMM computation. Conventional ADMM without the blockchain needs less than 0.1 ms per iteration and would complete the attack-free run (56 iterations) within a few milliseconds, whereas the same run takes 110 s on the chain. The cost of the blockchain is thus determined by the block period rather than by computation.

### 7.3. Large-Scale Evaluation and Ablation

Because the six-UAV network is a stress case, the main evaluation uses the 1000-UAV network, whose results Table 6 reports.

The results separate the roles of the two mechanisms. *Blockchain only* neutralizes the equivocating attacks and gives 0.35, 0.39, and 0.44 m for Byzantine deception and disruption with 100, 200, and 300 compromised UAVs, equal to the ideal WLS, because every equivocating sender is flagged and excluded; against attackers that commit a single false value (FDI and the single-value variants), however, it provides no protection, as stated in Section 4.2. *Screening only* handles consistent false values but cannot prevent equivocation, and for Byzantine deception it gives 0.47–0.99 m. By combining both mechanisms, the proposed defense reduces the FDI error from 23.6, 46.9, and 69.9 m to 0.49, 0.73, and 0.95 m (reductions of 97.9–98.6%), remains within 0.03 m of the ideal WLS under the equivocating attacks, and lowers the error under the single-value Byzantine and disruption attacks by 69–97%. For attacks with a single false value, its results are identical to those of screening only, because the blockchain has nothing to reject; the accuracy contribution of the blockchain thus consists of the reduction from 0.47–0.99 m to 0.37–0.45 m under equivocation. Without attacks, the screening increases the RMSE from 0.33 to 0.35 m, which is the cost of about 2% false alarms. Every secured run satisfies the convergence criterion, whereas all undefended disruption runs reach the iteration limit; in every configuration of Table 6, either all ten runs or none meet the criterion, except signed exchange alone under equivocating disruption with 100 compromised UAVs (four of ten). Because the defended runs under equivocating disruption meet this criterion before the first sign switch at iteration 100, we repeated all disruption configurations for a fixed horizon of 400 iterations without early stopping (Table 7). Under equivocating disruption, the RMSE of the proposed defense over the last complete switching cycle (iterations 201–400) equals its value before the first switch (0.37–0.45 m), because the excluded states never enter the iteration; under single-value disruption, it decreases from 0.80–1.55 m before the first switch to 0.64–1.07 m, and in every run, the largest RMSE after the first switch stays below the value before it.

For Byzantine deception with 100 compromised UAVs, signed exchange performs similarly to the blockchain (0.36 versus 0.35 m), because the 8-nearest-neighbor graph has many triangles in which conflicting messages can be compared. Its protection is weaker for disruption and degrades as compromised UAVs become denser, with a disruption RMSE of 0.72, 1.24, and 2.54 m for 100, 200, and 300 compromised UAVs, whereas the blockchain keeps it at the ideal-WLS level (0.35–0.44 m), because non-equivocation through the blockchain does not depend on common neighbors. With screening added, signed exchange comes close to the proposed defense. The two differ by at most 0.13 m, and the difference exceeds the confidence intervals only for equivocating disruption with 200 and 300 compromised UAVs (0.41 versus 0.45 m and 0.45 versus 0.58 m). The W-MSR baseline is consistently less accurate than the proposed defense at this scale and does not meet the convergence criterion. This also holds under a placement satisfying the F=1 local fault-count condition, in which 63–70 compromised UAVs are chosen such that no honest UAV has more than one compromised neighbor; the proposed defense reaches 0.37–2.39 m under FDI, single-value, adaptive, and equivocating attacks, versus 0.59–4.11 m for W-MSR (0.38 and 4.11 m under FDI), whose graph robustness we did not verify.

Table 8 lists the false-alarm and missed-detection rates, the iteration counts, and the number of conflicting records rejected by the blockchain per iteration for the proposed defense. For the equivocating attacks, the blockchain rejects 821, 1652, and 2470 conflicting records per iteration, and all states of flagged senders are excluded. For FDI, 5.9–6.5% of the compromised messages are accepted, mainly during the warm-up, and the false-alarm rate rises to 6–10% because honest UAVs near the attackers are temporarily displaced. By design, more than half of the messages of the adaptive attacker are accepted.

### 7.4. Calibration of the Screening Threshold

The results above rely on the calibrated screening rule of Section 5.4. Table 9 compares alternative rules for 200 compromised UAVs and shows that, without a warm-up (W=0), the residuals are dominated by the prior errors and 36–81% of the honest messages are rejected for κ between 3 and 2. The network then loses most of its cooperative information, and even without attacks the RMSE rises to 1.4–2.0 m, close to the 2.1 m of the priors alone. With a warm-up of 10 iterations, the false-alarm rate drops to 8.3%, 2.1%, and 0.4% for κ=2, 2.5, and 3. A longer warm-up of 30 iterations lowers the false alarms only slightly but admits more compromised messages during the warm-up (13–15% missed detections under FDI, compared with 6–8%). Smaller values of κ detect more attacks; for the single-value Byzantine attack, for example, the RMSE is 0.87, 1.28, and 1.90 m for κ=2, 2.5, and 3. We select κ by false-alarm calibration, fixing a budget of 5% and using the smallest tested κ whose bound $\exp(-\kappa^{2}/2)$ from Lemma 1 stays within this budget. This gives κ=2.5 (bound 4.4%, measured 2.1%), whereas κ=2 exceeds it (bound 13.5%, measured 8.3%); the bound is an attack-free reference near convergence, and the measured rates include the transient after the warm-up. A smaller threshold also limits how far an adaptive attacker can shift its false state, and κ=2 therefore yields a lower RMSE under every attack in Table 9 (for example 3.42 instead of 5.01 m under the adaptive attacker), at the cost of rejecting about four times as many honest messages. Applications that can tolerate more false alarms may therefore choose κ=2.

Clipping, used instead of replacement, retains a bounded part of each flagged state. Against consistent false states, this bounded part is applied in every iteration and accumulates, and the FDI error rises to 20.3 m, which is why the proposed defense uses replacement. The $\chi^{2}$ rule that accounts for the prior uncertainty at initialization yields thresholds of 5.3–6.6 m, which accept the FDI offsets entirely (RMSE 37.7–43.9 m). All rules in Table 9 nevertheless met the stopping criterion in every run (71–257 iterations on average, at most 344).

### 7.5. Adaptive Attacker and Remaining Vulnerability

**Figure 6:**
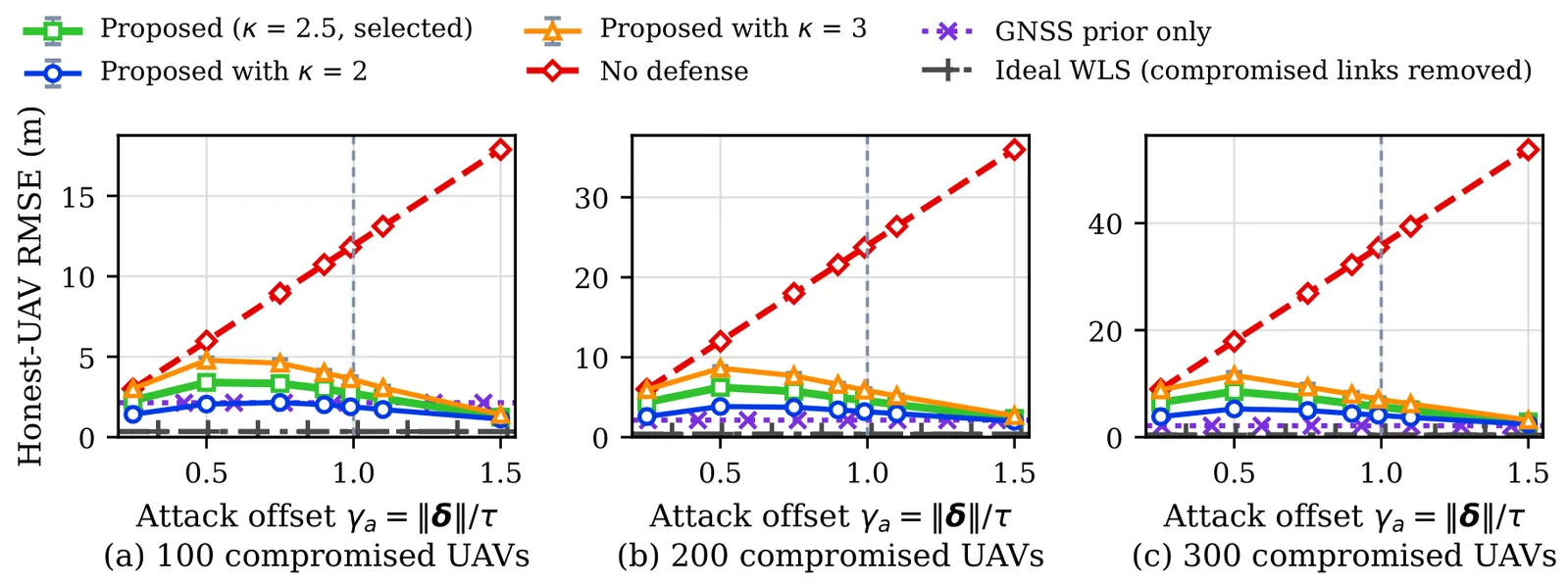
Honest-UAV RMSE in the simulated 1000-UAV network as a function of the offset of the adaptive attacker for 100, 200, and 300 compromised UAVs.

Unlike the attacks above, the adaptive attacker of Equation (6) attempts to keep its false states inside the screening regions of its targets and thereby probes the limit of the proposed defense. Figure 6 shows the honest-UAV RMSE as a function of its offset $\gamma_{a}$ (mean and 95% confidence interval over ten runs). The attack is most damaging when it stays well inside the screening region ($\gamma_{a}\approx 0.5$), where with κ=2.5 the RMSE reaches about 3.4, 6.2, and 8.5 m for 100, 200, and 300 compromised UAVs. Offsets above the threshold ($\gamma_{a}>1$) are increasingly detected and therefore less harmful. Without defense, the same attacker causes errors of 3–54 m. The defense therefore limits, but does not remove, the effect of a consistent false state within the threshold, and the residual error exceeds the accuracy of the priors alone. In agreement with Proposition 3, the error grows with κ and with the number of compromised neighbors. This is the main remaining vulnerability of the method in the considered setting, because a blockchain cannot certify the correctness of a single committed state, and a measurement check can only bound its influence.

### 7.6. Sensitivity to Noise Levels and Initial Errors

All results so far use the nominal noise levels of Table 3 and initial states at the priors; Table 10 varies both with 200 compromised UAVs. The method is effective when the injected offset is large compared with the threshold $\kappa \sigma_{y}$ and the priors are not much less accurate than the relative measurements, and it loses its benefit in two cases. When $\sigma_{y}=1$ m, the threshold (2.5 m) is close to the FDI offset (2.8 m), 29–45% of the compromised messages are accepted, and the RMSE approaches or exceeds that of the priors. When $\sigma_{g}/\sigma_{y}=12$, the 10-iteration warm-up is too short for the large prior errors; without attack the screening rejects 5.6% of honest messages, and under FDI the runs do not converge within 800 iterations and the RMSE (4.79 m) exceeds that of the priors (4.27 m). These cases agree with the dependence on $\kappa \sigma_{y}$ and $\sigma_{g}^{2}/\sigma_{y}^{2}$ in Proposition 3. Gaussian errors of up to $s_{0}=5$ m in the initial states (lower group of Table 10) change the RMSE by less than 0.05 m.

### 7.7. Scalability of the Blockchain

The preceding sections evaluate accuracy, whereas the cost of the blockchain at scale requires a separate measurement. We let 6 to 1000 UAV accounts submit one state record each simultaneously to the same six-validator network, five rounds per size, in the default configuration of the prototype and in an optimized configuration (Table 11). Each record of the first contract version (Section 7.2) is one signed transaction of 334 bytes that consumes about 279,000 gas, and no transaction failed in either configuration. A record of the current version has 306 bytes and needs about 257,000 gas, which raises the capacity of a 30-million gas block from 107 to 116 records, so that the latencies below are, if anything, conservative. The time per iteration runs from the start of the submission until all records are read back, as in the completion-based closing of the prototype, which a fixed-height schedule matches only with a period Δ at least this long, and the on-chain TPS divides the number of records by the time from the timestamp of the block preceding the first record to that of the block containing the last record (at least 1 s). In the default configuration, the 30-million gas limit admits 107–108 records per block, and all records are submitted to one node. An iteration then takes about 2 s up to 50 UAVs but 6.0, 10.2, and 19.3 s with 200, 500, and 1000 UAVs, whose records occupy 3.0, 5.6, and 10.4 blocks on average. The node logs show that the signers of many blocks had not yet received the records and sealed few or none, which halves the sustained throughput (about 55 TPS, computed from block timestamps with 1 s resolution), and the client needs about 9 s to sign and submit 1000 records on one computer.

The optimized configuration removes these bottlenecks without changing the smart contract. The chain is re-initialized with a 350-million gas limit (about 1250 records per block) and otherwise identical genesis parameters, every record is broadcast to all six validators, and the records are signed before the timed window, which emulates parallel signing on the UAVs (1.1 ms per record), whereas the default timing includes the signing of all records by one client. An iteration then takes 1.8–2.0 s up to 200 UAVs, 2.35 s with 500 UAVs, and 3.80 ± 0.26 s with 1000 UAVs, five times less than the default configuration with its different gas limit, submission, and timing boundary, and the on-chain throughput reaches about 900 TPS; the validators import a block with 800 records (223 million gas) in about 0.4 s, well within the 1 s block period. Of the remaining 3.8 s, about 2 s result from the sealing schedule of Clique, in which a record arriving during a block period is included two blocks later, and 1.3 s from reading all 1000 receipts through one client, which is an upper bound because each UAV reads only the records of its neighbors. During this test, one block was replaced by a competing one, and no record was lost.

Multiplying the measured iteration time by the 75–251 iterations of the defended simulations gives an estimated 4.8–15.9 min for a defended 1000-UAV localization, instead of 24–81 min. The load test contains one record per UAV; under equivocation, the conflicting records add load, for example about 1650 records of 85,679 gas per iteration with 200 compromised UAVs, which requires about one additional block. In terms of messages, each iteration carries one record per UAV, whereas peer-to-peer ADMM exchanges 2|E|≈9300 messages and signed exchange adds about 44,500 forwarded digests, and every Clique node executes about $2.8\times 10^{8}$ gas per iteration. These measurements hold for six validators on one host and do not establish performance over wireless links.

## 8. Discussion

This section discusses the latency of the blockchain and the applications it suits, its contribution and trade-off with latency, and extensions of the method.

**Latency and suitable applications:** The prohibitive latency of the prototype for moving UAVs results from its synchronous design, in which every ADMM iteration waits until the records of all UAVs are sealed in a block, which with the 1 s block period of Clique takes about 2 s. The defended ADMM therefore takes 3–4 min on the chain for six UAVs (Table 5) and an estimated 5–16 min for 1000 UAVs (Section 7.7), whereas its computation takes less than 1 ms per iteration. The framework is therefore best suited to applications in which accurate and verifiable position estimates matter more than latency, such as hovering formations, relay networks, and pre-mission calibration on the ground, periodic integrity checks of the positions while the onboard navigation controls the flight, and post-mission analysis based on the auditable record.

**Contribution of the blockchain and trade-off with latency:** The ablation shows that most of the accuracy gain comes from the screening and that the blockchain improves accuracy mainly where equivocation occurs. Because signed exchange combined with screening achieves nearly the same accuracy in our 8-nearest-neighbor networks (Section 7.3), the distinctive value of the blockchain lies in preventing equivocation in every iteration independently of the topology and in an auditable record of every submitted state and conflict, which supports later attribution of misbehaving UAVs; the latency is the price of this guarantee. Exchanging signed states directly and anchoring them on the chain once per localization would reduce the blockchain cost to a single round (about 3.8 s for 1000 UAVs) and retain most of the accuracy, but equivocation would then be detected only afterwards through the anchored record, and its detection within the iterations would depend on common neighbors. Likewise, each additional confirmation lowers the risk that a reorganization changes a record in use but adds one block period per iteration (Section 4.2).

**Extensions:** Several extensions beyond the scope of this paper can broaden the applicability of the method. For moving UAVs, the static two-dimensional model can be replaced by a three-dimensional motion model, with the ADMM warm-started from the previous estimates, which reduces the number of iterations per localization, and with a screening threshold that includes the motion uncertainty. Time-varying graphs and packet losses can be handled by asynchronous or event-triggered ADMM variants, in which a missing record is treated like an excluded state (Section 5.3). The iteration time can be reduced further by Byzantine-fault-tolerant blockchains with sub-second block intervals and deterministic finality, operated by a small committee of validators on separate devices, which future work will test over wireless links. Finally, adaptive thresholds based on the convergence state of each UAV and reputation mechanisms based on the on-chain conflict records can further limit the influence of adaptive attackers.

## 9. Conclusions

This paper presented a blockchain-assisted ADMM framework for distributed UAV localization under Byzantine deception, disruption, FDI, and adaptive attacks, in which a permissioned blockchain provides non-equivocation and accountability and a calibrated relative-measurement screening bounds the influence of consistent false states. A symmetric edge update makes the method independent of UAV labels and of computations by compromised neighbors, and, for honest neighbors that accept each other, the analysis establishes its convergence when the screening decisions stabilize and reject every compromised state, together with a bound on the deviation of any fixed point. In 1000-UAV simulations, the proposed defense reduces the FDI error by 97.9–98.6% and keeps the error under equivocating attacks within 0.03 m of the ideal WLS estimate, whereas an adaptive attacker that attempts to evade the threshold still causes 3.4–8.5 m, which marks its main remaining vulnerability. The six-node prototype reproduces the simulations to within 2 µm, and an iteration takes about 2 s with six UAVs and, with a raised gas limit, 3.8 s with 1000 UAVs, suiting quasi-static networks, integrity checks, and post-mission analysis.

## Figures and Tables

**Table 1:** Comparison of closely related technical studies and the proposed method.

Work	Core Method	Self-Localization	Distributed	Byzantine-Resilient	Non-Equivocation Layer	Physical-Consistency Check
Zeng et al. [31]	RIS-assisted collaborative localization	Yes	Yes	No	No	No
Ding et al. [32]	Set-membership localization	Yes	Yes	No	No	No
Cheng et al. [33]	Distributed RSS fusion	No	Yes	No	No	No
Zhang et al. [34]	Cooperative fusion localization	No	Yes	No	No	No
Michieletto et al. [8]	Robust sensor fusion under spoofing	Yes	Yes	No	No	Yes
Liu et al. [36]	Robust topology recovery	No	Yes	No	No	No
LeBlanc et al. [37]	W-MSR-resilient consensus	No	Yes	Yes	No	No
Sundaram and Gharesifard [38]	Resilient distributed optimization	No	Yes	Yes	No	No
Fang et al. [41]	Byzantine-resilient decentralized gradient descent	No	Yes	Yes	No	No
This work	Blockchain-assisted ADMM with measurement screening	Yes	Yes	Yes	Yes	Yes

**Table 3:** Experimental parameters of the simulations and configuration of the blockchain prototype. Italicized headings distinguish the localization experiments from the blockchain prototype configuration.

Parameter	Value
*Localization experiments*	
Noise standard deviations	$\sigma_{g}=1.5$ m (absolute priors), $\sigma_{y}=0.5$ m (relative measurements); sensitivity analysis with $\sigma_{g}\in \left\{0.5,1.5,3\right\}$ m and $\sigma_{y}\in \left\{0.25,0.5,1\right\}$ m
ADMM	ρ=1; $\epsilon_{p}=\epsilon_{d}=10^{-4}$ m (RMS); at most 800 iterations; $x_{i}^{\left(0\right)}=g_{i}$, copies at the priors, duals zero
Screening	κ=2.5 (τ=1.25 m), warm-up W=10, replacement; compared with κ∈{2,2.5,3}, W∈{0,10,30}, clipping, and the $\chi^{2}$ rule
Byzantine deception	$\gamma_{b}=1.9$ m; base direction (1,−0.6)/∥(1,−0.6)∥ rotated by ${\left(\left(131j+17i\right)\;\mathrm{mod}\;360\right)}^{\circ }$ for receiver *i*
Disruption	$\gamma_{s}=14$ m; base direction (1,−0.8)/∥(1,−0.8)∥, receiver-dependent rotation as above; L=100
FDI	$\gamma_{f}=2.8$ m; direction (1,−0.6)/∥(1,−0.6)∥
Adaptive attacker	$\gamma_{a}=0.9$ (sweep 0.25–1.5); direction as for FDI
W-MSR baseline	F=1
Metrics	Honest-UAV RMSE (RMSE over honest UAVs); false-alarm rate (flagged honest messages/honest messages) and missed-detection rate (accepted compromised messages/compromised messages) at honest receivers
Reference estimates	GNSS prior only; ideal WLS (WLS with all links of compromised UAVs removed)
Monte Carlo	10 runs per configuration (seeds 7–16); 95% confidence intervals
Simulation software	Python 3.12.3 (figure/table reproduction); NumPy 2.4.4, SciPy 1.17.1, pandas 3.0.2, Matplotlib 3.10.8
*Blockchain prototype*	
Client	Geth v1.13.14-stable (Go 1.21.6), Windows build
Consensus and finality	Clique proof of authority [52]; M=6 signers (the six UAV accounts); period 1 s; a signer may seal at most one of every 4 consecutive blocks; no per-block quorum certificate; records are read after one confirmation
Chain identifier/network identifier	12345/123454321
Block gas limit	30,000,000 gas (107–108 records per block); 350,000,000 gas in the optimized load test
Smart contract and libraries	Solidity 0.8.0 (EVM target istanbul, $10^{-6}$ m resolution); Python 3.10.0, NumPy 2.0.2, SciPy 1.15.3, pandas 2.2.2, Web3.py 8.0.0
Peering	Boot node plus direct static peering of all six nodes over the loopback interface

**Table 4:** Honest-UAV RMSE (m) in the simulated six-UAV network for each attack and defense, reported as the mean ± 95% confidence interval over ten realizations. Bold values indicate the lowest mean RMSE in each row, including ties. Italicized text identifies the compromised-UAV placement scenarios. ^†^ indicates runs that reached the 800-iteration limit.

	Defense				
Attack	No Defense	Blockchain Only	Screening Only	W-MSR Baseline	Proposed
*Compromised $U_{1}$, $U_{4}$: prior only 2.13 m, attack-free WLS 1.29 m, ideal WLS 1.50 m*					
No attack	**1.22 ± 0.38**	**1.22 ± 0.38**	**1.22 ± 0.38**	1.58 ± 0.45 ^†^	**1.22 ± 0.38**
Byzantine (equivocating)	10.40 ± 0.56	4.11 ± 0.58	1.83 ± 0.41	1.60 ± 0.60 ^†^	**1.50 ± 0.50**
Byzantine (single value)	11.66 ± 0.54	11.66 ± 0.54	2.13 ± 0.60	**1.71 ± 0.56** ^†^	2.13 ± 0.60
Disruption (equivocating)	62.77 ± 0.71 ^†^	25.03 ± 5.56	2.00 ± 0.52	**1.61 ± 0.51** ^†^	1.83 ± 0.45
FDI	16.39 ± 0.93	16.39 ± 0.93	**1.72 ± 0.58**	2.78 ± 0.75 ^†^	**1.72 ± 0.58**
Adaptive	10.51 ± 1.16	10.51 ± 1.16	5.37 ± 1.34	**1.84 ± 0.48** ^†^	5.37 ± 1.34
*Two compromised UAVs placed at random: prior only 2.03 m, attack-free WLS 1.24 m, ideal WLS 1.27 m*					
No attack	**1.22 ± 0.38**	**1.22 ± 0.38**	**1.22 ± 0.38**	1.58 ± 0.45 ^†^	**1.22 ± 0.38**
Byzantine (equivocating)	7.19 ± 2.05	3.11 ± 1.11	1.52 ± 0.62	1.61 ± 0.41 ^†^	**1.27 ± 0.34**
Byzantine (single value)	7.46 ± 2.53	7.46 ± 2.53	**1.74 ± 0.63**	1.81 ± 0.35 ^†^	**1.74 ± 0.63**
Disruption (equivocating)	45.53 ± 12.39 ^†^	17.16 ± 8.91	1.85 ± 0.38	1.65 ± 0.35	**1.58 ± 0.33**
FDI	17.25 ± 3.86	17.25 ± 3.86	**1.68 ± 0.66**	3.43 ± 1.63 ^†^	**1.68 ± 0.66**
Adaptive	9.89 ± 1.52	9.89 ± 1.52	4.67 ± 1.76	**2.41 ± 0.89** ^†^	4.67 ± 1.76

**Table 5:** Blockchain-integrated six-UAV runs on the Clique prototype compared with simulations of the same configurations.

Attack	Defense	Iterations	RMSE, Chain (m)	RMSE, Simulation (m)	Maximum Difference (µm)	Equivocation Events	Time per Iteration (s)	Total Time (s)
No attack	Proposed	56	1.683	1.683	0.6	0	1.97	110
FDI	Blockchain only	94	16.145	16.145	0.6	0	1.96	184
FDI	Proposed	106	2.069	2.069	0.3	0	1.95	207
Byzantine (equivocating)	Blockchain only	83	4.247	4.247	0.7	166	1.97	163
Byzantine (equivocating)	Proposed	95	2.070	2.070	1.1	190	1.99	189
Byzantine (single value)	Proposed	104	2.681	2.681	1.7	0	1.96	204
Disruption (equivocating)	Proposed	120	2.070	2.070	0.6	240	1.96	235
Adaptive	Proposed	81	5.682	5.682	0.4	0	1.96	159

**Table 6:** Honest-UAV RMSE (m) in the simulated 1000-UAV network, reported as the mean ± 95% confidence interval over ten runs. Bold values indicate the lowest mean RMSE in each row, including ties. Italicized text identifies the compromised-UAV scenarios. ^†^ indicates runs that did not satisfy the stopping criterion within 800 iterations; their reported RMSE is averaged over the final 100 iterations.

	Defense						
Attack	No Defense	Blockchain Only	Screening Only	Signed Exchange Only	Signed Exchange + Screening	W-MSR Baseline	Proposed
*100 compromised UAVs (prior only 2.14 m; attack-free WLS 0.33 m; ideal WLS 0.35 m)*							
No attack	**0.33 ± 0.02**	**0.33 ± 0.02**	0.35 ± 0.01	**0.33 ± 0.02**	0.35 ± 0.01	0.46 ± 0.02 ^†^	0.35 ± 0.01
Byzantine (equivocating)	1.20 ± 0.13	**0.35 ± 0.02**	0.47 ± 0.02	0.36 ± 0.01	0.37 ± 0.02	0.73 ± 0.05 ^†^	0.37 ± 0.02
Byzantine (single value)	3.23 ± 0.45	3.23 ± 0.45	**0.68 ± 0.09**	3.23 ± 0.45	**0.68 ± 0.09**	1.49 ± 0.13 ^†^	**0.68 ± 0.09**
Disruption (equivocating)	7.24 ± 0.69 ^†^	**0.35 ± 0.02**	0.56 ± 0.04	0.72 ± 0.10 ^†^	0.39 ± 0.02	1.28 ± 0.13 ^†^	0.37 ± 0.02
Disruption (single value)	20.16 ± 2.32 ^†^	20.16 ± 2.32 ^†^	**0.64 ± 0.04**	20.16 ± 2.32 ^†^	**0.64 ± 0.04**	3.81 ± 0.61 ^†^	**0.64 ± 0.04**
FDI	23.60 ± 0.19	23.60 ± 0.19	**0.49 ± 0.05**	23.60 ± 0.19	**0.49 ± 0.05**	10.72 ± 0.32 ^†^	**0.49 ± 0.05**
Adaptive	10.73 ± 0.12	10.73 ± 0.12	**3.01 ± 0.12**	10.73 ± 0.12	**3.01 ± 0.12**	4.98 ± 0.11 ^†^	**3.01 ± 0.12**
*200 compromised UAVs (prior only 2.14 m; attack-free WLS 0.33 m; ideal WLS 0.39 m)*							
No attack	**0.33 ± 0.02**	**0.33 ± 0.02**	0.35 ± 0.01	**0.33 ± 0.02**	0.35 ± 0.01	0.46 ± 0.02 ^†^	0.35 ± 0.01
Byzantine (equivocating)	1.56 ± 0.21	**0.39 ± 0.01**	0.71 ± 0.05	0.43 ± 0.02	0.42 ± 0.01	1.03 ± 0.13 ^†^	0.41 ± 0.02
Byzantine (single value)	4.39 ± 0.41	4.39 ± 0.41	**1.28 ± 0.11**	4.39 ± 0.41	**1.28 ± 0.11**	2.51 ± 0.20 ^†^	**1.28 ± 0.11**
Disruption (equivocating)	9.61 ± 1.05 ^†^	**0.39 ± 0.01**	0.76 ± 0.04	1.24 ± 0.14 ^†^	0.45 ± 0.02	3.68 ± 0.58 ^†^	0.41 ± 0.02
Disruption (single value)	27.51 ± 2.14 ^†^	27.51 ± 2.14 ^†^	**0.89 ± 0.04**	27.51 ± 2.14 ^†^	**0.89 ± 0.04**	10.41 ± 1.21 ^†^	**0.89 ± 0.04**
FDI	46.87 ± 0.39	46.87 ± 0.39	**0.73 ± 0.13**	46.87 ± 0.39	**0.73 ± 0.13**	28.30 ± 0.60 ^†^	**0.73 ± 0.13**
Adaptive	21.58 ± 0.26	21.58 ± 0.26	**5.01 ± 0.17**	21.58 ± 0.26	**5.01 ± 0.17**	11.63 ± 0.26 ^†^	**5.01 ± 0.17**
*300 compromised UAVs (prior only 2.14 m; attack-free WLS 0.33 m; ideal WLS 0.44 m)*							
No attack	**0.33 ± 0.02**	**0.33 ± 0.02**	0.35 ± 0.01	**0.33 ± 0.02**	0.35 ± 0.01	0.46 ± 0.02 ^†^	0.35 ± 0.01
Byzantine (equivocating)	1.91 ± 0.12	**0.44 ± 0.02**	0.99 ± 0.05	0.57 ± 0.03	0.48 ± 0.02	1.44 ± 0.10 ^†^	0.45 ± 0.02
Byzantine (single value)	5.71 ± 0.45	5.71 ± 0.45	**1.77 ± 0.06**	5.71 ± 0.45	**1.77 ± 0.06**	4.12 ± 0.26 ^†^	**1.77 ± 0.06**
Disruption (equivocating)	11.85 ± 0.66 ^†^	**0.44 ± 0.02**	0.97 ± 0.06	2.54 ± 0.26 ^†^	0.58 ± 0.02	6.51 ± 0.49 ^†^	0.45 ± 0.02
Disruption (single value)	35.76 ± 2.32 ^†^	35.76 ± 2.32 ^†^	**1.07 ± 0.03**	35.76 ± 2.32 ^†^	**1.07 ± 0.03**	21.29 ± 1.88 ^†^	**1.07 ± 0.03**
FDI	69.90 ± 0.34	69.90 ± 0.34	**0.95 ± 0.09**	69.90 ± 0.34	**0.95 ± 0.09**	48.13 ± 0.84 ^†^	**0.95 ± 0.09**
Adaptive	32.25 ± 0.33	32.25 ± 0.33	**6.26 ± 0.34**	32.25 ± 0.33	**6.26 ± 0.34**	18.97 ± 0.38 ^†^	**6.26 ± 0.34**

**Table 7:** Fixed-horizon disruption runs in the simulated 1000-UAV network (400 iterations without early stopping, switching interval L=100; means over ten runs, with 95% confidence intervals for the mean RMSE; the maximum is taken within each run and averaged over the runs).

		Proposed Defense				Blockchain Only	No Defense
Disruption	Compromised UAVs	RMSE at It. 100 (m)	Mean RMSE 201–400 (m)	Max. RMSE 101–400 (m)	First Tol. Iteration	Mean RMSE 201–400 (m)	Mean RMSE 201–400 (m)
Equivocating	100	0.37	0.37 ± 0.02	0.37	75	0.35 ± 0.02	7.23 ± 0.69
	200	0.41	0.41 ± 0.02	0.41	75	0.39 ± 0.01	9.61 ± 1.06
	300	0.45	0.45 ± 0.02	0.45	77	0.44 ± 0.02	11.9 ± 0.7
Single-value	100	0.80	0.64 ± 0.04	0.79	206	20.2 ± 2.3	20.2 ± 2.3
	200	1.22	0.89 ± 0.04	1.20	231	27.5 ± 2.1	27.5 ± 2.1
	300	1.55	1.07 ± 0.03	1.53	251	35.8 ± 2.3	35.8 ± 2.3

**Table 8:** Detection statistics of the proposed defense for 1000 simulated UAVs (10-run means). Italicized headings identify the compromised-UAV scenarios.

Attack	False Alarms (%)	Missed Detections (%)	Iterations	Rejected Conflicting Records per Iteration
*100 compromised UAVs*				
No attack	2.1	–	74	0
Byzantine (equivocating)	2.0	0.0	75	821
Byzantine (single value)	6.0	18.0	164	0
Disruption (equivocating)	2.0	0.0	75	821
Disruption (single value)	21.9	4.9	206	0
FDI	5.9	5.9	201	0
Adaptive	5.3	57.0	143	0
*200 compromised UAVs*				
No attack	2.1	–	74	0
Byzantine (equivocating)	1.9	0.0	75	1652
Byzantine (single value)	10.8	21.0	188	0
Disruption (equivocating)	1.9	0.0	75	1652
Disruption (single value)	30.0	4.4	231	0
FDI	8.4	6.5	217	0
Adaptive	8.4	55.1	184	0
*300 compromised UAVs*				
No attack	2.1	–	74	0
Byzantine (equivocating)	1.7	0.0	77	2470
Byzantine (single value)	14.8	22.8	190	0
Disruption (equivocating)	1.7	0.0	77	2470
Disruption (single value)	36.0	4.0	251	0
FDI	9.7	6.2	227	0
Adaptive	10.4	52.9	224	0

**Table 9:** Comparison of screening rules in the simulated 1000-UAV network with 200 compromised UAVs in terms of false alarms, missed detections, and honest-UAV RMSE (means over ten runs). Bold texts indicate our proposed setting and the corresponding performance.

	No Attack		FDI Attack		Byzantine Attack (Single Value)	Adaptive Attack
Screening Rule	False Alarms (%)	RMSE (m)	RMSE (m)	Missed Detections (%)	RMSE (m)	RMSE (m)
Replace, κ=2, W=0	80.6	2.01	2.11	4.0	2.13	2.09
Replace, κ=2.5, W=0	54.1	1.68	2.05	7.0	2.12	2.57
Replace, κ=3, W=0	36.1	1.43	2.00	10.0	2.15	3.62
Replace, κ=2, W=10	8.3	0.38	0.67	5.6	0.87	3.42
Replace, κ=2.5, W=10	**2.1**	**0.35**	**0.73**	**6.5**	**1.28**	**5.01**
Replace, κ=3, W=10	0.4	0.33	0.94	7.7	1.90	6.59
Replace, κ=2, W=30	6.3	0.38	0.68	13.1	0.83	3.42
Replace, κ=2.5, W=30	1.5	0.34	0.67	13.3	1.26	5.06
Replace, κ=3, W=30	0.2	0.33	0.90	14.5	1.94	6.56
Clip, κ=2.5, W=10	1.7	0.33	20.30	11.1	3.38	15.43
$\chi^{2}$, α=0.01 (τ≈6.6 m)	0.0	0.33	43.94	97.7	4.42	34.43
$\chi^{2}$, α=0.05 (τ≈5.3 m)	0.1	0.33	37.72	91.8	4.42	27.36

**Table 10:** Sensitivity of the proposed defense to the noise levels and initial errors in the simulated 1000-UAV network with 200 compromised UAVs (κ=2.5, W=10; means over ten runs). Italics distinguish the evaluated scenarios; ^†^ indicates runs reaching the 800-iteration limit, with RMSE averaged over the final 100 iterations.

				No Attack		FDI Attack		
$\sigma_{g}$ (m)	$\sigma_{y}$ (m)	Initial Error $s_{0}$ (m)	Threshold τ (m)	RMSE (m)	False Alarms (%)	RMSE (m)	Missed Detections (%)	Prior-Only RMSE (m)
*Noise levels (initial error $s_{0}=0$)*								
0.5	0.25	0	0.625	0.16	2.1	0.20	3.3	0.71
0.5	0.5	0	1.25	0.27	2.0	0.31	11.1	0.71
0.5	1	0	2.5	0.43	1.9	0.55	45.2	0.71
1.5	0.25	0	0.625	0.21	3.5	1.24	5.6	2.14
1.5	0.5	0	1.25	0.35	2.1	0.73	6.5	2.14
1.5	1	0	2.5	0.59	1.9	2.00	29.7	2.14
3	0.25	0	0.625	0.33	5.6	4.79 ^†^	11.1	4.27
3	0.5	0	1.25	0.42	2.9	2.89	8.4	4.27
3	1	0	2.5	0.69	2.2	6.57	28.7	4.27
*Initial errors ($\sigma_{g}=1.5$ m, $\sigma_{y}=0.5$ m)*								
1.5	0.5	0	1.25	0.35	2.1	0.73	6.5	2.14
1.5	0.5	2	1.25	0.35	2.4	0.69	6.2	2.14
1.5	0.5	5	1.25	0.36	3.2	0.75	6.5	2.14

**Table 11:** Measured blockchain latency, block usage, and throughput versus the number of UAVs submitting in the same iteration, in the default (30-million gas limit, one node) and optimized (350-million gas limit, broadcast) configurations; five rounds per size.

	Default Configuration				Optimized Configuration			
UAVs	Time per Iteration (s)	Blocks with Records	On-Chain TPS	Maximum Block Size (kB)	Time per Iteration (s)	Blocks with Records	On-Chain TPS	Maximum Block Size (kB)
6	1.91 ± 0.16	1.0	6	2.6	1.92 ± 0.10	1.0	6	2.6
25	2.00 ± 0.07	1.0	25	9.0	1.96 ± 0.04	1.0	25	9.0
50	2.16 ± 0.43	1.2	45	17.3	1.93 ± 0.04	1.0	50	17.3
100	3.40 ± 0.82	1.8	50	34.0	1.89 ± 0.10	1.0	100	34.0
200	5.95 ± 0.03	3.0	40	36.4	1.80 ± 0.14	1.0	200	67.4
500	10.18 ± 1.16	5.6	55	36.4	2.35 ± 0.23	1.0	500	167.6
1000	19.25 ± 0.97	10.4	55	36.4	3.80 ± 0.26	1.2	900	334.6

## Data Availability

The simulation and blockchain code, the smart contract, the experiment scripts, all result files (including per-run results of the Monte Carlo experiments and the on-chain logs), and the scripts that generate every figure and data table of this article from these result files are available at https://github.com/Mainul333/UAV-localization-ADMM-security-blockchain (accessed on 31 October 2026).
